# Provenance‐Specific Chilling and Forcing Requirements Shape Spring Phenology in Three European Temperate Tree Species

**DOI:** 10.1111/gcb.70851

**Published:** 2026-04-10

**Authors:** Zhaofei Wu, Manuel G. Walde, Ilka Beil, Marcin Klisz, Yann Vitasse

**Affiliations:** ^1^ Swiss Federal Institute for Forest, Snow and Landscape Research WSL Birmensdorf Switzerland; ^2^ Experimental Plant Ecology University of Greifswald Greifswald Germany; ^3^ Dendrolab IBL, Department of Silviculture and Genetics of Forest Trees Forest Research Institute Sekocin Stary Poland; ^4^ Oeschger Centre for Climate Change Research University of Bern Bern Switzerland

**Keywords:** budburst success, budburst timing, chilling accumulation, forcing requirement, temperate trees, winter warming

## Abstract

Global warming is altering spring phenology in temperate forests, with important consequences for tree survival, growth, and ecological interactions. However, temperature requirements for dormancy release and budburst vary among populations adapted to different climatic conditions, complicating predictions of spring phenology across broad geographic regions. Here, we quantified the chilling and forcing requirements of three deciduous tree species ( *Fagus sylvatica* , *Quercus robur* , and *Tilia cordata* ) using four provenances per species spanning a latitudinal gradient from Spain to Poland. Saplings were overwintered under either ambient or warmed open‐top chambers and were transferred monthly from November to February to a climate chamber under constant forcing conditions. We found that reduced chilling due to earlier transfer substantially delayed budburst, with *T. cordata* showing the highest chilling requirement, followed by *F. sylvatica* , whereas *Q. robur* exhibited the lowest. We detected both co‐ and counter‐gradient patterns of genetic variation in budburst timing. In *Q. robur* and, to a lower extent, in *T. cordata* , Polish provenances budburst later than Spanish ones, while German and Swiss populations were intermediate (co‐gradient). In contrast, *F. sylvatica* showed the opposite pattern with the Spanish provenance tending to budburst latest and the Polish one earliest (counter‐gradient). These differences likely reflect genetic differentiation in chilling and forcing requirements among provenances, likely driven by variation in frost risk at their sites of origin. Importantly, insufficient chilling significantly reduced budburst success by 25%–85% across species, with the strongest effect in *T. cordata* , where success fell below 10% in saplings transferred in November or December across all provenances, potentially constraining canopy development and impairing growth and reproduction. These findings underscore the critical role of winter chilling in regulating budburst timing and canopy development, as well as provenance‐specific adaptation, suggesting that species adapted to low chilling might be candidates for assisted migration under rapid climate warming.

## Graphical abstract

Winter chilling is essential for dormancy release and spring budburst in temperate trees. Using a climate‐controlled experiment with three species and four European provenances spanning a broad latitudinal gradient, we show that reduced chilling delays budburst and decreases budburst success, with strong species‐ and provenance‐specific differences. These findings highlight the critical role of winter chilling in regulating spring phenology and canopy development and imply an increasing risk of incomplete budburst under warmer winters, particularly at the warmest margins of species distributions.

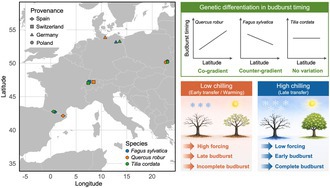

## 1 Introduction

In temperate forests, the timing of spring leaf‐out plays a critical role in tree survival (Chuine and Beaubien 2001), growth and carbon uptake (Keenan et al. 2014), and influences key biotic interactions within ecosystems (Vitasse et al. 2021). In response to climate warming, earlier spring phenology has been observed in temperate and boreal regions (Fu et al. 2015; Vitasse et al. 2022; Wu et al. 2026). Although earlier leaf‐out may enhance carbon sequestration by extending the growing season (Keenan et al. 2014), it can also increase vulnerability to late spring frosts (Zohner, Mo, Sebald, and Renner 2020) and summer drought through earlier water demand (Meier et al. 2021), both of which can impair tree health and productivity. When certain thresholds are exceeded, these species‐specific shifts can disrupt intra‐ and interspecific interactions and trigger cascading effects on food web and ecosystem functioning (Kharouba et al. 2018). This highlights the importance of accurately predicting spring phenology under future climate scenarios to improve projections of ecosystem responses, guide assisted migration strategies, and maintain forest resilience in a rapidly changing climate.

Temperate tree species typically undergo two dormancy phases: winter endodormancy and spring ecodormancy (Lang et al. 1987). During endodormancy, buds require exposure to cold temperatures—a process known as *chilling accumulation* (Wang et al. 2020). Once sufficient chilling is fulfilled, trees transition to ecodormancy, during which buds begin to accumulate heat (often quantified as forcing units such as growing degree‐days, GDD), while remaining responsive to additional chilling that reduces the heat required for budburst (Cannell and Smith 1986). However, it remains unclear when dormancy is released (Laube et al. 2014; Walde et al. 2024)—that is, when buds begin to accumulate forcing—and whether this timing is fixed or varies across years due to differences in dormancy depth. This is especially important for phenology modeling under projected warmer winters in the temperate zone, where reduced chilling may delay phenological development or even become insufficient for proper development. Insufficient chilling can also reduce the proportion of buds that successfully develop (Walde et al. 2022), leading to incomplete canopy development, reduced photosynthetic surface area and diminished reproductive performance (Rhie et al. 2012). In addition, insufficient chilling may also restrict growth in the subsequent growing season (Man et al. 2017). While this phenomenon is well‐documented in fruit trees, where chilling requirements are crucial for yield and quality (Wenden et al. 2016), it has far less been studied in forest tree species (but see Baumgarten et al. 2021; Man et al. 2017). As winters become milder in most temperate regions, understanding how insufficient chilling affects phenology and bud development is critical for predicting forest productivity and regeneration under climate change (Kreyling 2010).

There is growing interest regarding whether tree populations from warmer regions may be better adapted to future warmer climates (Wang et al. 2025; Wu et al. 2023). Most provenance trials focus on tolerance to heat and drought, whereas only a few studies investigate differences in chilling and forcing requirements (Kramer et al. 2017)—despite their importance for predicting whether leaf‐out aligns with late spring frost risk. Southern provenances may have evolved lower chilling requirements to fully release dormancy, allowing earlier forcing accumulation and spring growth (Liang 2016). However, our understanding of how chilling and forcing requirements vary between provenances and the extent to which these differences are genetically controlled is limited (Vitasse et al. 2009; Walde et al. 2022; Wu et al. 2022). This gap partially reflects the difficulty of quantifying these processes under controlled conditions, as most studies rely on field observations or limited experiments rather than systematic tests of provenances under strict controlled climate chambers.

**FIGURE 1:**
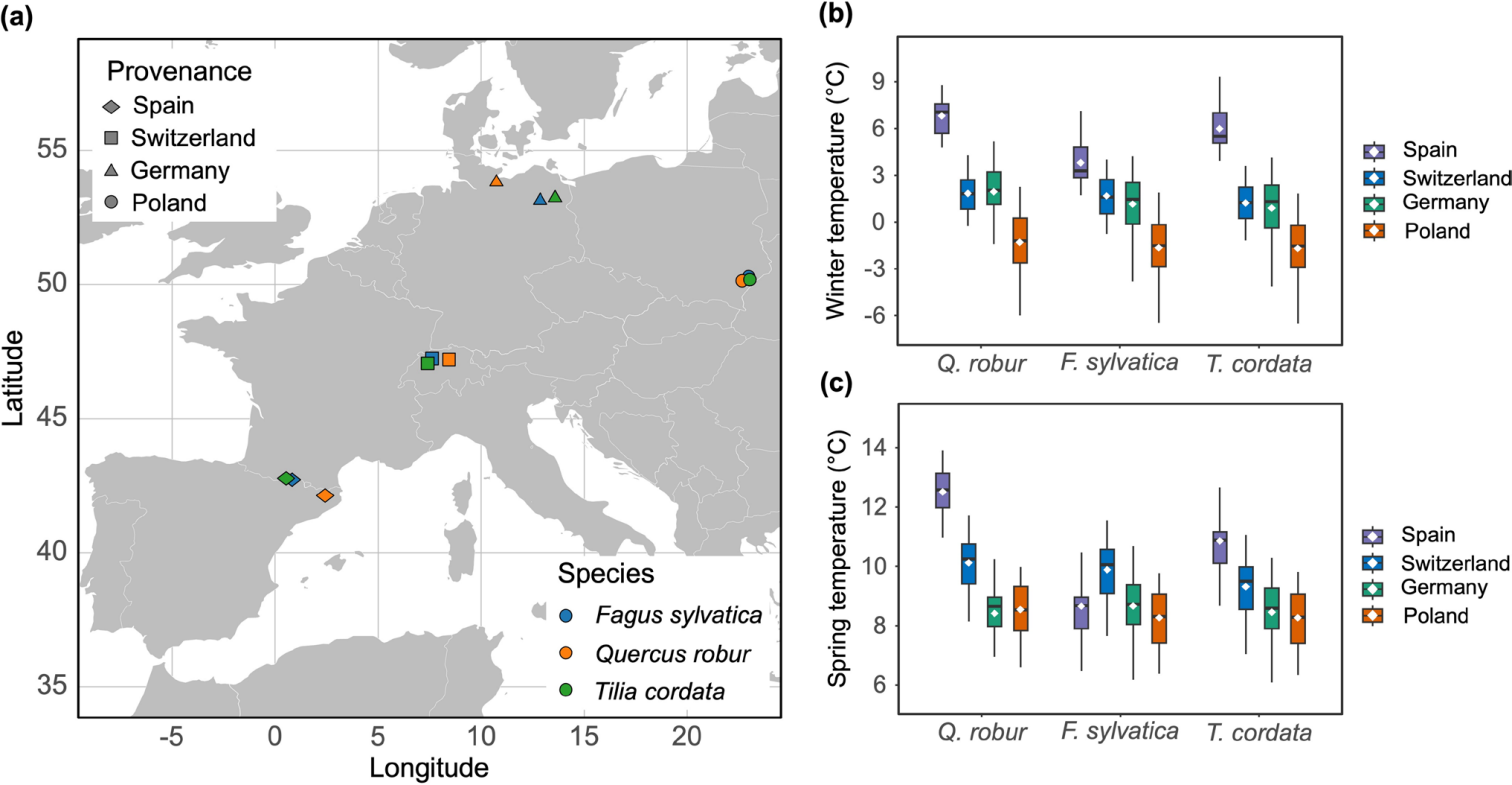
Locations and background climate of the sapling provenances. (a) Geographic locations of the sapling provenances for different tree species. (b, c) Multi‐year mean winter (December–February, b) and spring (March–May, c) temperatures at the sapling provenance sites during 1991–2020. Boxplots show the first quartile, median, and third quartile, as well as the minimum and maximum values within 1.5 times the interquartile range. The white square indicates the mean value.

In this study, we experimentally quantified the chilling and forcing requirements of three deciduous tree species— *Fagus sylvatica* L., *Quercus robur* L. *and Tilia cordata* Mill.—each represented by four provenances spanning a latitudinal gradient from Spain to Poland (Figure 1a). Potted saplings were kept during winter under either ambient conditions or +5°C warming in open‐top chambers (OTCs) and were transferred monthly from November to February into climate chambers under constant forcing conditions (20°C). This approach enabled us to assess how chilling accumulation affects the dormancy release and budburst timing. Moreover, this approach allowed us to test whether southern provenances require less chilling and to assess how chilling and forcing interact in regulating budburst timing and success across a large climatic and geographic gradient. We tested the following two hypotheses: (1) The timing of budburst among provenances follows a co‐gradient pattern with their native climate, that is, populations from colder origins flushing later than those from warmer regions; however, opposite pattern may occur in species whose southern provenances exhibit higher forcing requirements (Liang 2016). (2) Reduced chilling delays budburst and lowers budburst success, particularly in species and provenances with high chilling requirement (Walde et al. 2022), potentially limiting canopy development and subsequent growth.

## 2 Material and Methods

### 2.1 Study Species and Provenances

We investigated the effects of winter chilling on spring budburst timing and success in three European tree species: *Fagus sylvatica* L. (beech), *Quercus robur* L. (oak), and *Tilia cordata* Mill. (small‐leaved lime). To assess variation among populations from contrasting climates, we purchased one‐year‐old individuals of each species from four provenances (Spain, Switzerland, Germany, and Poland) spanning a latitudinal gradient from 42°10′ N to 53°37′ N and a longitudinal gradient from 0°41′ E to 19°58′ E (Figure 1a). The saplings were transferred to Birmensdorf, Switzerland during the winter 2023/2024 (Polish in December, Swiss in January, and German and Spanish in February), repotted into 4 L plastic pots in February 2024 and kept outside until the experiment began in November 2024. The pots were filled with a mixture of two‐thirds sandy, peaty substrate and one‐third compost soil. Fertilizer was applied during the growing seasons to ensure adequate nutrient supply.

Substantial variation in sapling height was observed at the end of 2024 (i.e., after one growing season in natural conditions), especially for *F. sylvatica* and *T. cordata* (Table S1). For *F. sylvatica* , the tallest individuals originated from Poland, followed by Switzerland, Germany, and Spain. *T. cordata* likewise exhibited the greatest height in Poland, followed by Spain, Switzerland, and Germany (Table S1).

### 2.2 Experimental Design and Treatments

The experiment was conducted at the WSL Research Institute in northeastern Switzerland (47°21′38″ N, 8°27′16″ E; 550 m a.s.l.), located in a temperate climate zone. The mean annual temperature recorded at the nearby Zurich/Kloten weather station from 1991 to 2020 was 9.8°C, and the mean annual precipitation was approximately 1000 mm, with the wettest months occurring in late spring and summer. Historical monthly mean temperatures from November to February were 4.7°C, 1.6°C, 0.9°C, and 1.6°C, respectively.

#### 2.2.1 Winter Warming

To investigate the effect of winter warming on bud development, the saplings were placed in open‐top chambers (OTCs) on November 1, 2024 under two temperature treatments prior to their transfer to forcing conditions: (1) a control treatment in OTCs exposed to ambient air temperature (no‐warming treatment), and (2) a warming treatment in OTCs where air temperature was maintained 5°C above ambient conditions with heating units (5°C warming treatment, see more details in Grossiord et al. 2022). Four OTCs were used—two for the no‐warming treatment and two for the 5°C warming treatment. Saplings were arranged in a randomized block design, with each treatment (i.e., transfer dates) distributed evenly among the four OTCs.

**FIGURE 2:**
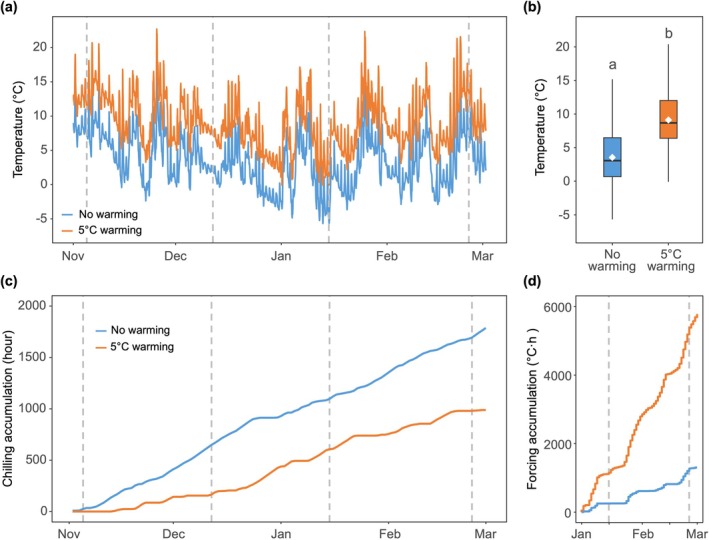
Temperature variation and the accumulation of chilling and forcing in the no‐warmed and warmed open‐top chambers. (a) Daily air temperature in the controlled and warmed chambers from November 1, 2024, to February 28, 2025. Dashed lines indicate the four transfer dates. The no‐warming treatment indicates the open‐top chambers without warming; the 5°C‐warming treatment refers to the open‐top chambers with a 5°C increase above ambient temperature. (b) Mean air temperature of the two winter warming treatments in the open‐top chambers. Boxplots show the first quartile, median, and third quartile, as well as the minimum and maximum values within 1.5 times the interquartile range. The white square represents the mean value. Different letters denote significant differences between winter temperature based on a two‐sided *t*‐test (*p* < 0.05). (c) Chilling accumulation in the open‐top chambers from the Chilling Hour model since 1 November 2024. (d) Growing degree hours above 5°C in the open‐top chambers from January 1, 2025 until the last transfer of the saplings.

HOBO sensors (HOBO MX2203, Onset Computer Corporation) were used to record air temperature at 15‐min intervals at plant height. No significant temperature differences were found among chambers within the same temperature treatment during winter; therefore, they were pooled for the subsequent analysis. Daily air temperatures in the no‐warming treatment closely followed ambient conditions but were on average 0.8°C higher (2.7°C ± 0.1°C vs. 3.5°C ± 0.1°C). The 5°C warming treatment was, on average, 5.6°C warmer than the no‐warming treatment, with a mean temperature of 9.1°C ± 0.1°C from November to February (Figure 2a,b).

#### 2.2.2 Chilling Exposure and Forcing Conditions

Saplings were transferred monthly into the same climate chambers to experience constant forcing conditions (20°C, 12‐h photoperiod) on four dates between November and February (Figure 2): November 5, 2024; December 12, 2024; January 15, 2025; and February 25, 2025. These transfer dates represented different levels of winter chilling, with earlier transfer dates corresponding to lower chilling accumulation (Walde et al. 2022; Wu et al. 2023). No winter‐warming treatment was applied for the November transfer date, representing the dormancy depth of the saplings at the onset of the chilling phase of the experiment.

In total, the experiment comprised 7 treatments (2 winter conditions × 3 transfer dates +1 transfer date at the beginning of the experiment). Each treatment included six saplings per tree species and provenance, resulting in a total of 504 potted saplings (3 species × 4 provenances × 7 treatments × 6 saplings per treatment). The climate chambers were equipped with halogen lamps (Philips MASTER TL‐D) providing a photosynthetic photon flux density (PPFD) of approximately 50 μmol m^−2^ s^−1^. Saplings were watered at 1–2 week intervals to avoid water limitation. None of the *T. cordata* saplings transferred into the climate chamber in November achieved budburst by April, so the chamber temperature was reduced to 3°C on April 10, 2025 (when all other saplings had already achieved budburst), for 2 weeks and subsequently to 2°C on May 22, 2025, for 3 weeks to increase chilling exposure. The experiment was stopped 10 months after the saplings were placed in the chambers (September, 2025), although the remaining *T. cordata* saplings were still alive.

### 2.3 Phenology Monitoring

Bud development of each sapling was monitored twice per week. Budburst was defined as the date when the first bud scale opened and the leaf became partially visible (Vitasse 2013; Wu et al. 2025). Time to budburst was defined as the number of days from the transfer date to the recorded budburst date.

To assess the effect of chilling on leaf development, we recorded the number of buds that achieved budburst and the total number of buds on each sapling (Table S1) 10 days after the first bud of the sapling reached budburst (Walde et al. 2024). Budburst success was calculated as the percentage of buds that achieved budburst to the total number of buds per sapling (Wu et al. 2022).

### 2.4 Data Analysis

Long‐term daily air temperature data (1991–2020) were obtained from the E‐OBS dataset for each provenance site (Cornes et al. 2018). For each site, the typical near‐surface air temperature lapse rate (0.6°C per 100 m) was used to correct for differences in elevation between the provenance sites and the extracted climate data (Kollas et al. 2014). The multi‐year mean winter (December–February) and spring (March–May) temperatures were calculated to represent the background climate conditions. Among the four sapling provenances, Spain represented the warmest climate, with winter temperatures of 5.5°C ± 0.19°C, followed by Switzerland (1.5°C ± 0.14°C) and Germany (1.3°C ± 0.21°C), whereas Poland represented the coldest provenance (−1.6°C ± 0.22°C; Figure 1b). A similar spatial pattern was observed for spring temperatures; however, for *F. sylvatica* sites, Switzerland was the warmest (Figure 1c). Frost risk at each native site was quantified for every day of the year as the occurrence of daily minimum temperature < 0°C over the period 1991–2020 (Walde et al. 2022).

Chilling accumulation was quantified as chilling hours, defined as the number of hours with temperatures between 0°C and 7.2°C from 1 November 2024 to each transfer date (Luedeling 2012), with each hour at temperatures between these thresholds contributing one chilling hour. This model has been widely used in phenological researches (Wang et al. 2020; Wu et al. 2023). Accumulated chilling under different winter warming treatments and transfer dates is presented in Figure 2c. Forcing accumulation was calculated as growing degree hours (GDH), represented by the cumulative sum of temperatures above 5°C at the time of budburst (Hunter and Lechowicz 1992). This included forcing accumulated in the open‐top chambers prior transfer since 1 January 2025 (Figure 2d) and in the climate chambers after the transfer date until budburst. We used the forcing requirement obtained from saplings transferred in February (no‐warming treatment) as the minimum forcing requirement, and the difference in forcing requirement between the February and December transfers as a proxy for chilling requirement.

Two‐sided *t*‐tests were used to evaluate the effects of transfer date and winter warming on budburst timing, as well as differences among species and sapling provenances (Wu et al. 2023). A binomial generalized linear model (GLM) was used for budburst success, as it represents proportional data ranging from 0 to 1. To evaluate the contribution of environmental and genetic factors to the forcing requirement and budburst success, a multi‐factor analysis of variance (ANOVA) was performed for each species with provenance, transfer date and winter temperature treatment as explanatory variables (Equation 1):

(1) $$\begin{array}{c} \text{Forcing requirement}/\text{Budburst success}\sim \text{Transfer date}\\ \times \text{Winter warming}\times \text{Sapling provenance} \end{array}$$

The most parsimonious models were identified by stepwise model selection based on Akaike's Information Criterion (AIC), starting from a null model. For each best‐fit model, we extracted the sum of squares (SS) for each factor and calculated the proportion of variance explained as the percentage of the total SS. The same analysis was conducted by combining the three species and including interactions between species and provenance, transfer date, and winter temperature treatment. The selected models are summarized in Table S2.

Furthermore, we investigated the effects of chilling hours on forcing requirements and budburst success using a quadratic nonlinear regression model (Forcing requirement = a + b × Chilling + c × Chilling^2^) and a Gompertz model (Budburst success = a × exp.(−b × exp.(−c × Chilling))), respectively. All statistical analyses were conducted in R 4.2.2.

## 3 Results

### 3.1 Effects of Sapling Provenance and Chilling Accumulation on Budburst Timing

Overall, 473 out of 504 saplings (94%) achieved budburst during the experiment (Table S3). However, there were pronounced differences between species. Almost all *F. sylvatica and Q. robur* saplings reached budburst across all treatments. In contrast, 67%–92% of *T. cordata* saplings transferred in December achieved budburst, while nearly 100% did so in the January and February transfers. For saplings transferred in November, none of the *T. cordata* saplings reached budburst by April, and only four did so after an additional 5 weeks of artificial chilling (Table S3). These individuals were excluded from subsequent analyses.

**FIGURE 3:**
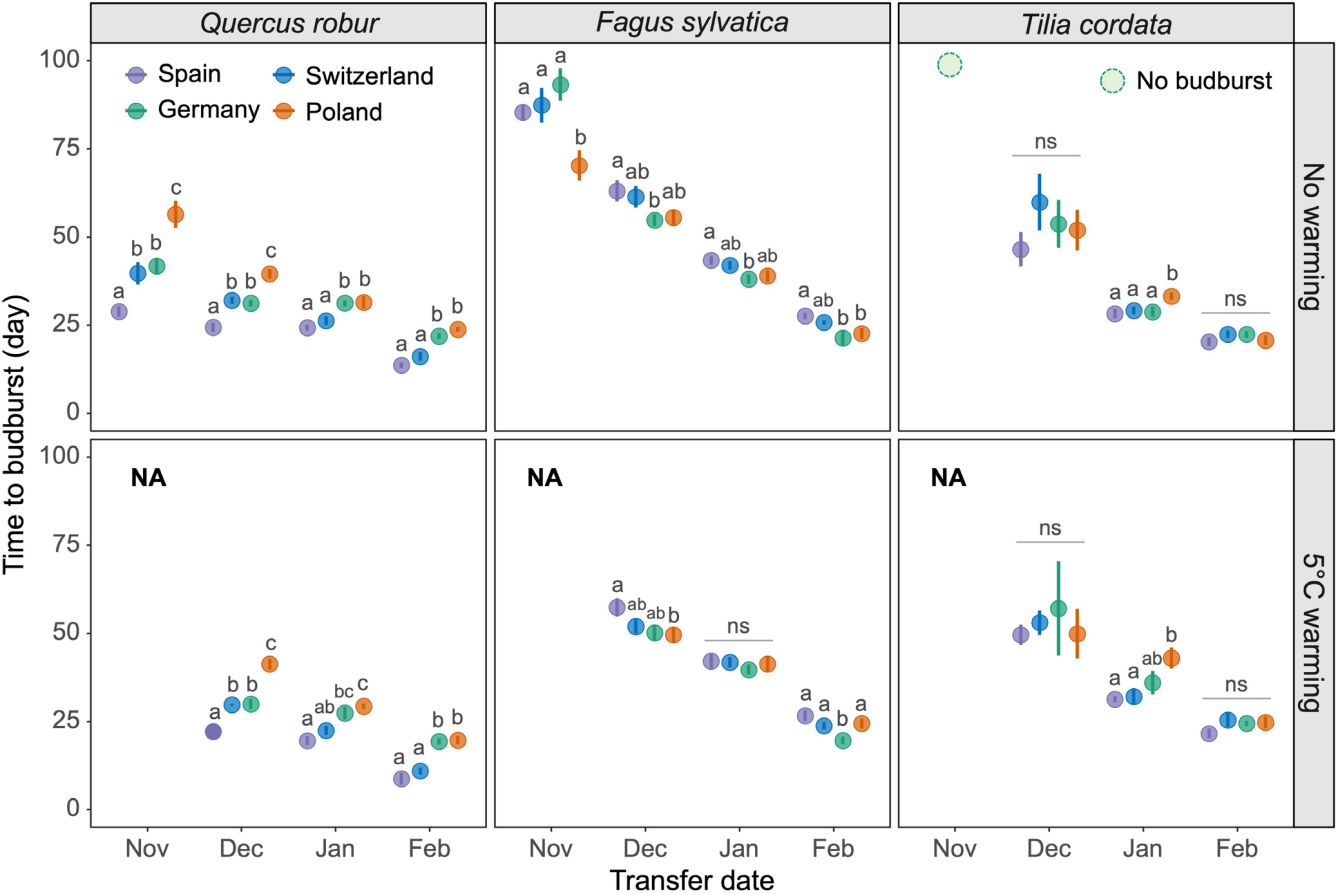
Effect of sapling provenances on the time to budburst under different transfer dates and winter temperature treatments. Time to budburst is represented as the days to budburst after being transferred to the 20°C climate chamber. The transfer dates represented different levels of winter chilling accumulation, with earlier transfers corresponding to lower chilling levels. The no‐warming treatment indicates the open‐top chambers without warming; the 5°C warming treatment refers to the open‐top chambers with a 5°C increase above ambient temperature. Each data point represents the mean ± se of six individual saplings per provenance for each transfer date and winter temperature treatment combination. The green dashed points indicate saplings that had not yet reached budburst by the end of the experiment but were still alive. Different letters denote significant differences between sapling provenances based on a two‐sided *t*‐test (*p* < 0.05); “ns” indicates no statistically significant difference among the sapling provenances.

The budburst timing varied among sapling provenances within species and followed a genetic cline according to the latitude of origin for *F. sylvatica* and *Q. robur* (Figures 3 and S1a). Across all treatments, *Q. robur* saplings from Spain exhibited the shortest time to budburst (20.2 ± 1.1 days), followed by Switzerland (25.2 ± 1.5 days), Germany (28.9 ± 1.2 days), and Poland (34.5 ± 1.9 days), consistent with a co‐gradient genetic cline along the winter–spring temperature of their regions of origin. In contrast, *F. sylvatica* showed a counter‐gradient genetic cline, with saplings from warmer origins flushing later, while significant differences were mainly observed between the Spain and Germany/Poland provenances (Figure 3). No significant provenance difference was detected for *T. cordata* , although saplings from Poland showed significantly delayed budburst under intermediate chilling exposure (i.e., transfer in January), indicating a weak co‐gradient genetic cline (Figure 3).

The transfer date, representing winter chilling accumulation, strongly affected budburst timing (Figures 3 and S1b). The highest exposure to chilling temperatures (February transfer) significantly shortened the time to budburst by an average of 60.1 and 24.9 days compared to the lowest exposure to chilling temperatures (November transfer) for *F. sylvatica* and *Q. robur* , respectively, and by 30.0 days compared to the December transfer for *T. cordata* (Figure S1b). Variation in budburst timing among provenances and species increased under insufficient chilling (Figures 3 and S2). Winter warming also had a significant effect on the time to budburst, shortening it for *Q. robur* and *F. sylvatica* , but delaying it for *T. cordata* , particularly in the January and February transfers (Figure S3).

### 3.2 Effects of Sapling Provenance and Chilling Accumulation on Forcing Requirement

**FIGURE 4:**
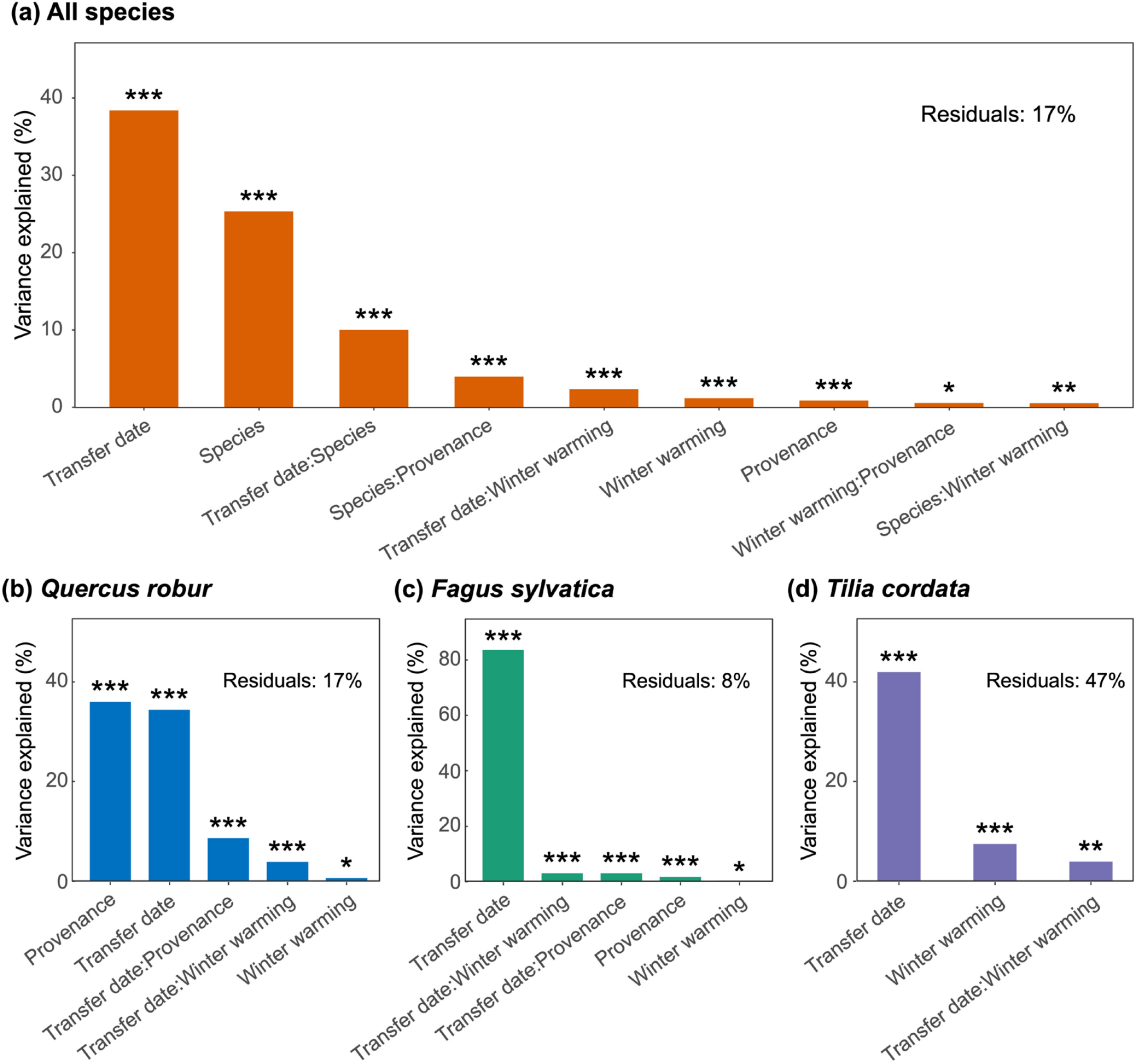
Interactive effects of sapling provenance, transfer date, and winter temperature treatment on forcing requirements for each tree species. The contribution of each variable to forcing requirement was assessed using a multi‐factor analysis of variance (ANOVA), with forcing requirement as the response variable and the interaction among sapling provenance, transfer date, winter temperature treatment, and tree species as explanatory variables. The most parsimonious model was identified via stepwise model selection based on Akaike's Information Criterion (AIC), starting from a null model. The residual represents the proportion of variance not explained by the selected variables. Asterisks indicate statistical significance based on *F*‐tests from ANOVA: ***p < 0.001; **p < 0.01; *p < 0.05.

Consistent patterns were observed for the forcing requirement to achieve budburst (Figures S4–S6). Across all treatments and species, the transfer date explained most of the variation in forcing requirement (38.4%), followed by inter‐species differences (25.3%, Figure 4a). The effect of transfer date on forcing requirement differed among tree species, being most pronounced in *F. sylvatica* (83.6%, Figure 4c), followed by *T. cordata* (41.9%, Figure 4d) and *Q. robur* (34.4%, Figure 4b). Specifically, forcing requirement decreased significantly with later transfer dates: for the February transfer, *Q. robur* showed a reduction of 39% (6101°C·h) and *F. sylvatica* a reduction of 61% (19,189°C·h) compared to the lowest chilling treatment (November), while *T. cordata* exhibited a reduction of 40% (7991°C·h) compared to the December transfer (Figure S4b). In addition, for *Q. robur* saplings, provenance explained a similar proportion of variance as transfer date (36.0%), and both sapling provenance and winter warming interacted with transfer date (Figure 4b).

Winter warming generally increased forcing requirements, particularly in the January and February transfers, by 7% (1155°C·h) and 27% (3833°C·h) for *F. sylvatica* , and by 21% (3037°C·h) and 36% (5130°C·h) for *T. cordata* , respectively. In contrast, winter warming decreased the forcing requirement by 11% (2377°C·h) for *F. sylvatica* in the December transfer, whereas no significant differences were observed for *Q. robur* or *T. cordata* (Figures S5 and S6).

### 3.3 Effects of Sapling Provenance and Chilling Accumulation on Budburst Success

**FIGURE 5:**
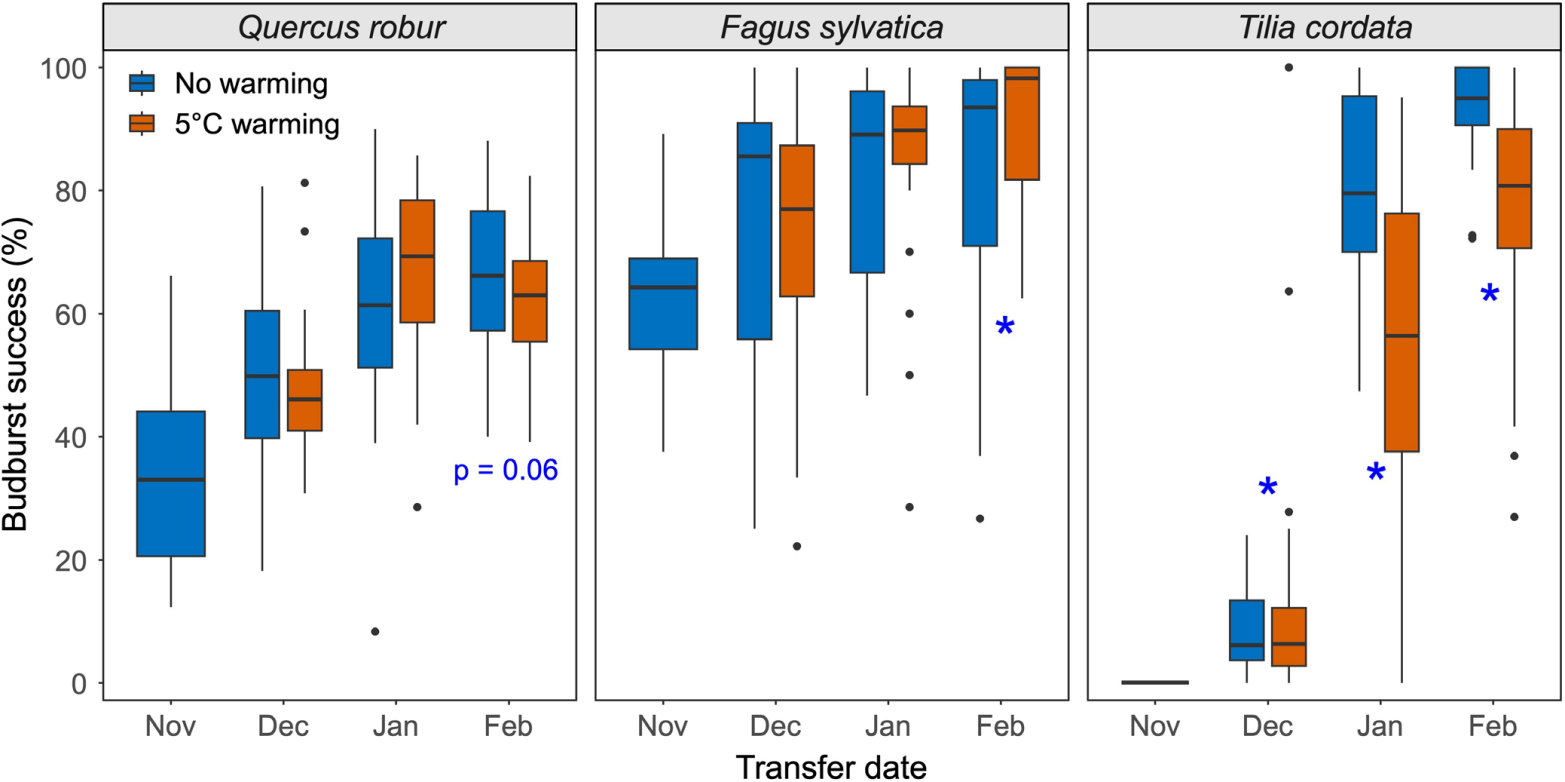
Budburst success under different transfer dates and winter temperature treatments for each tree species. Budburst success was calculated as the percentage of buds that achieved budburst to the total number of buds per sapling. The no‐warming treatment indicates the open‐top chambers without warming; the 5°C‐warming treatment refers to the open‐top chambers with a 5°C increase above ambient temperature. Boxplots show the first quartile, median, and third quartile, as well as the minimum and maximum values within 1.5 times the interquartile range. Asterisks (*) denote significant differences between winter temperature treatments based on a two‐sided *t*‐test (*p* < 0.05); other pairwise comparisons were not statistically significant.

Across all treatments, budburst success was highest in *F. sylvatica* (78%), followed by *Q. robur* (55%) and *T. cordata* (50%, Figures 5 and S7). Among them, transfer date explained the greatest proportion of variation in budburst success (34.1%), followed by species (18.6%, Figure S8a). Budburst success increased significantly with later transfer dates for all species, but the magnitude of the increase differed among species (Figures 5, S7b and S8b–d). Transfer dates explained the largest proportion of variation in budburst success in *T. cordata* (77.0%), followed by *Q. robur* (38.6%) and *F. sylvatica* (16.9%). The budburst success of *Quercus robur* was affected by transfer date but not by winter warming and provenance, increasing from 33.8% for saplings transferred in November to 64.4% in February (Figures S7b and S8b). *T. cordata* showed the most pronounced chilling response, with budburst success rising from 0% for the November transfer to 84.8% for the February transfer. Winter warming reduced *T. cordata* budburst success by 23.2% (January transfer) and 16.8% (February transfer), but increased it by 7% (December transfer, Figures 5 and S9). In *F. sylvatica* , the budburst success increased from 62.5% for saplings transferred in November to 87.1% in February, and winter warming increased it by 7% for saplings transferred in February (Figures 5 and S7b). However, provenance explained more variation in budburst success (28.2%) than transfer date for *F. sylvatica* , with Swiss and Polish saplings showing the highest budburst success of 89.0% (Figures S7a and S8c). In addition, provenance also interacted with transfer date to influence budburst success (6.4%).

### 3.4 Effects of Chilling Exposure on Forcing Requirements and Budburst Success

**FIGURE 6:**
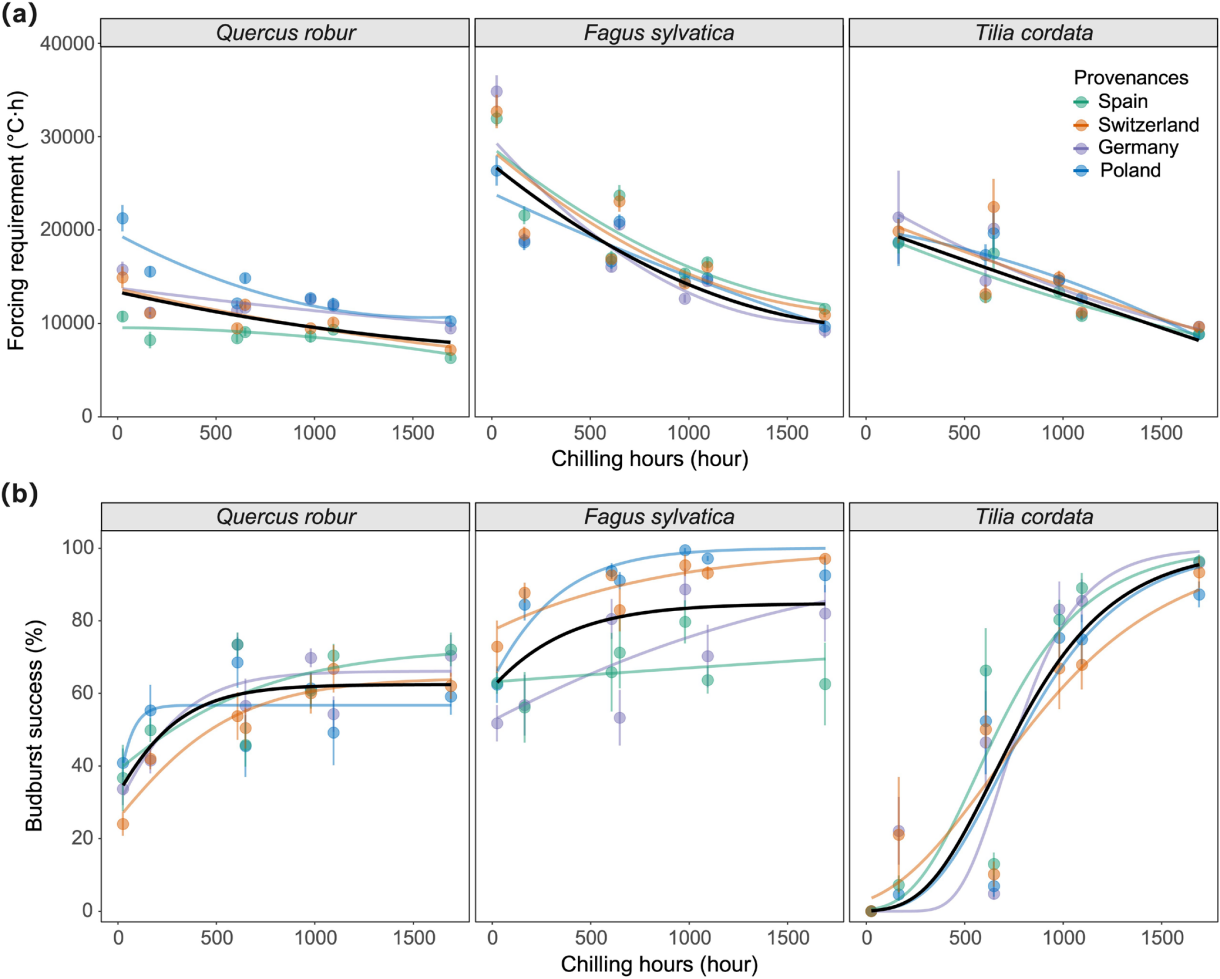
Effect of chilling exposure on forcing requirement (a) and budburst success (b) across different species and provenances. Chilling hours in the open‐top chambers was quantified as the total number of hours with mean air temperatures between 0°C and 7.2°C from 1 November 2024 to each transfer date. Lines represent model predictions, with forcing requirement fitted using a quadratic nonlinear regression model and budburst success fitted using a Gompertz model. Budburst success was calculated as the percentage of buds that achieved budburst to the total number of buds per sapling. Colored lines represent different provenances, and the black line shows the mean fitted response across all provenances.

Across all species and provenances, forcing requirement for budburst declined significantly with increasing chilling accumulation, particularly in *T. cordata* and *F. sylvatica* (Figure 6a). In contrast, the forcing requirement in *Q. robur* decreased slightly with chilling, and the Spanish provenance appeared insensitive to chilling.

Budburst success increased significantly with chilling accumulation across all species and provenances, especially in *T. cordata* , where it increased from 0 to nearly 100% (Figure 6b). In *F. sylvatica* and *Q. robur* , budburst success also increased with increasing chilling and remained at the highest level beyond 500–600 chilling hours. For *Q. robur* from Poland, budburst success stabilized once chilling accumulation reached ~200 h, whereas chilling had no effect on *F. sylvatica* from Spain.

## 4 Discussion

Our experiment demonstrates the critical role of winter chilling in regulating budburst timing and its success rate, with significant inter‐ and intra‐specific differences. Along the studied geographical gradient in Europe, provenances of *Q. robur* , and to a lesser extent *T. cordata*, from northern regions exhibited higher chilling and forcing requirements, showing a co‐gradient variation. In contrast, *F. sylvatica* showed a counter‐gradient genetic cline. Reduced exposure to chilling temperatures simulated by early transfer to forcing conditions markedly delayed budburst, with winter warming amplifying this effect during the latest transfer in January and February. Moreover, chilling deficits drastically reduced budburst success, especially in *T. cordata* , where almost none of the saplings transferred in November achieved budburst. These findings underscore the crucial role of winter chilling in regulating spring phenology and canopy development, as well as differences among species and provenances, providing critical insights to support decisions on the selection of suitable provenances and species for climate adaptation strategies.

### 4.1 Spatial Variation in Chilling and Forcing Requirements for Budburst

Genetic differentiation in phenology between tree populations reflects adaptations to local climatic conditions or to biotic interactions (e.g., herbivory, pathogens, competition with neighboring plants) (Savolainen et al. 2007), but can also arise through non‐adaptive processes such as genetic drift or founder effects (Petit et al. 2003). Our experiment highlighted later budburst in northern populations of *Q. robur* , reflecting a co‐gradient variation (Liang 2016). Such co‐gradient variation in budburst timing has been widely observed along latitudinal and elevational gradients (Alberto et al. 2013; McGee 1974; Vitasse et al. 2009), and is often attributed to higher chilling requirements in cold‐adapted provenances, reflecting their adaptation to the risk of late spring frosts (Liang 2016). Our estimation of provenance‐specific forcing and chilling requirements for saplings support that provenances of *Q. robur* from colder climates indeed exhibited greater chilling requirement than those from warmer conditions, while forcing requirement to budburst under maximal chilling exposure was relatively uniform (Figure S10a). We further analyzed provenance‐specific frost probabilities and found that saplings from Poland exhibit the highest probability to encounter late‐spring frost in their climate of origin, whereas Spain had the lowest, especially for the *Q. robur* site (Figure S11a), which further supports our frost‐avoidance hypothesis. Consequently, *Q. robur* may be under natural selection for higher chilling and forcing thresholds in colder regions with higher probability of late spring frosts (Lamichhane 2021; Zohner, Mo, Renner, et al. 2020).

In contrast, the opposite pattern was observed for *F. sylvatica* , that is, earlier flushing for provenances from colder climates, consistent with previous findings for the same species (Gömöry and Paule 2011; Vitasse et al. 2009) and for a couple of other species such as black spruce (Guo et al. 2022). Our results suggest that *F. sylvatica* from warmer climates require greater forcing to trigger budburst (Figure S10b), resulting in delayed budburst when grown under common conditions (Liang 2016). In contrast, since the growing season is shorter at high latitudes, plants generally have lower forcing thresholds to maximize seasonal growth (Hänninen 2016; Liang 2019). This is consistent with *F. sylvatica* 's late‐flushing strategy, which minimizes frost risk (Sangüesa‐Barreda et al. 2021). Although forcing requirements of *T. cordata* increased in northern populations for saplings transferred to forcing conditions in January, no spatial variation was observed in other treatments. This relatively uniform response across provenances suggests limited genetic control in dormancy regulation, which is likely due to *T. cordata* 's exceptionally high chilling requirement.

Consequently, both co‐gradient and counter‐gradient patterns can emerge when genotypes are placed in the same environment, depending on species‐specific frost‐avoidance strategies and chilling‐forcing interactions. Populations from colder climates may budburst earlier due to lower forcing requirements, or later due to higher chilling requirements (Liang 2016). The former leads to a counter‐gradient pattern as found for *F. sylvatica* , whereas the latter produces a co‐gradient pattern, as found for *Q. robur* . These results highlight the need for caution when combining large datasets across broad geographic ranges, as genetic adaptations may influence phenological responses and reduce model accuracy.

### 4.2 Effect of Chilling Accumulation on Forcing Requirement to Budburst

Earlier transfer dates significantly increased forcing requirement and delayed budburst timing, confirming that insufficient chilling delays spring phenological development (Laube et al. 2014; Walde et al. 2022; Wu et al. 2023). This effect was strongest in *F. sylvatica* and *T. cordata* , indicating that these two species are more sensitive to chilling and thus more vulnerable to potential chilling decline under further winter warming. In contrast, *Q. robur* likely met its chilling requirements as early as December, demonstrating its low chilling requirement (Baumgarten et al. 2021; Walde et al. 2022). Interestingly, the chilling effect in *Q. robur* was more pronounced in saplings from the coldest provenance (Poland). This interaction between chilling requirement and provenance may reflect genetic adaptations allowing populations growing in colder environments to not budburst too early and thus avoid potential spring frost damage.

Winter warming also affected budburst timing, by advancing it in *Q. robur* (species with low chilling requirement) in saplings transferred in January and February since these saplings were already sensitive to warming before the transfer and accumulated some degree days in the warming OTCs. However, winter warming increased the forcing requirement of *T. cordata* (species with high chilling requirement), probably because a 5°C warmer temperature throughout winter elevated ambient temperatures above the optimal range for chilling, resulting in insufficient chilling which increased the forcing requirement (Murray et al. 1989). It may also be because outdoor warming is less effective at promoting budburst when chilling accumulation is incomplete and/or photoperiods are short, leading to the apparent increase in forcing requirements. This effect was more pronounced in species with higher chilling requirements, such as *F. sylvatica* and *T. cordata* . However, the upper threshold of chilling efficiency of temperate tree species remains poorly understood (Baumgarten et al. 2021; Zhang et al. 2026), and further experiments are needed to accurately quantify at the species and provenance level, particularly as future winters may increasingly exceed these thresholds in the warmest part of their distribution.

Interestingly, warming before the December transfer reduced the forcing requirements of *F. sylvatica* and, to a lesser extent, *Q. robur* . Since dormancy induction in temperate trees requires both declining daylength and cold temperatures (Heide 2011; Maurya and Bhalerao 2017), elevated December temperatures may have delayed endodormancy onset and/or reduced dormancy depth (Beil et al. 2021). However, winter warming had no significant effect on the forcing requirement of *Q. robur* (except for February transfer), likely due to its low chilling requirement (Baumgarten et al. 2021; Murray et al. 1989). These findings highlight a critical phase in bud dormancy regulation, where buds transition from endodormancy to ecodormancy by accumulating a certain amount of chilling and eventually budburst after reaching a certain accumulation of heat (Beil et al. 2021; Fu et al. 2020). However, although buds become sensitive to forcing before chilling requirements are fully met, they remain responsive to additional chilling, which continues to lower forcing requirements (Wang et al. 2023), especially for species with high chilling requirements. In addition, although the widely used Chilling Hours model defines chilling units as temperatures between 0°C and 7.2°C, the exact lower and upper thresholds and whether chilling accumulation is constant or dynamic remain uncertain (Baumgarten et al. 2021). Further climate‐controlled experiments are therefore required to determine this phase transition and to identify species‐specific chilling thresholds and efficiencies (Chuine et al. 2016; Zhang et al. 2026).

### 4.3 Effect of Insufficient Chilling on Budburst Success and Potential Canopy Development

Insufficient chilling not only delayed budburst timing but also reduced budburst success across all species, which is consistent with previous studies (Baumgarten et al. 2021; Walde et al. 2022, 2024). This indicates that chilling deficits can impair bud development (Petri and Leite 2004; Zhang et al. 2021), likely by disrupting physiological processes required for the transition from dormancy to active growth, such as preventing the degradation of ABA. This effect shows species‐specific susceptibilities (Man et al. 2017), which was most pronounced in *T. cordata* , where none of the saplings transferred in November budburst after exposure to forcing conditions until April. Moreover, winter warming also significantly reduced budburst success of *T. cordata* , even in the latest transfer in February, suggesting that the projected warmer winters in Central and Eastern Europe may affect its development. Although later transfer dates significantly increased the budburst success of *Q. robur* , no significant difference was observed between the January and February transfers, indicating that the chilling requirement of oak saplings was likely fulfilled by January. Notably, the budburst success of *Q. robur* remained relatively low compared to the other species—reaching only about 70% even under the February treatment—which could be due to its large number of dormant buds (Table S1), which can open quickly after defoliation caused by frost or herbivores (Baumgarten et al. 2023). For *F. sylvatica* , sufficient chilling increased budburst success significantly, although success rates remained above 50% across all transfer dates. This indicates that although chilling accumulation is important for dormancy release, *F. sylvatica* reacts more resilient to chilling deficits than *T. cordata* .

These results imply that insufficient chilling may constrain canopy development, potentially reducing photosynthetic capacity and productivity (Petri and Leite 2004; Zhang et al. 2021). Impaired bud development may also influence reproductive performance by limiting flower initiation and reducing floral abundance, as commonly observed in fruit trees (Petri and Leite 2004). However, directly linking budburst success and tree growth and vitality remains understudied. Future experimental studies incorporating measurements of leaf area development and biomass accumulation would help clarify how chilling limitations translate into broader ecosystem consequences.

## 5 Conclusion

Our study highlights the critical role of winter chilling in regulating budburst timing and success, with substantial interspecific and provenance‐level differences along a broad latitudinal gradient in Europe. Species with high chilling requirements, such as *Tilia cordata* , may face an increased risk of delayed budburst and reduced leaf area under future warmer winter conditions, especially in the warmest parts of their distribution, where winter temperatures approach the upper limit of chilling efficiency. Current dynamic vegetation models often overlook species‐specific chilling requirements and dormancy transitions, which our results show can strongly affect budburst timing and success. Incorporating these mechanisms is therefore critical for improving predictions of canopy development and carbon cycling under future climate change scenarios. Long‐term field monitoring and physiological measurements across diverse climates, complemented by manipulative experiments, are needed to improve our understanding of dormancy regulation in temperate deciduous tree species. Integrating these insights into climate adaptation and forest management will be essential for reforestation or assisted migration and for maintaining ecosystem function and stability under climate change.

## Author Contributions

Y.V., M.G.W., and I.B. conceived the study and designed the experiment. Z.W. and Y.V. conducted the experiment. Z.W. analyzed the data. Z.W. and Y.V. led the manuscript writing, with input from M.G.W., I.B., and M.K. M.G.W. potted and maintained the saplings prior to the experiment. I.B. and M.K. assisted with the experiment preparation. All authors discussed the results and reviewed the manuscript.

## Funding

This work was supported by Swiss National Science Foundation, 315230_192712, 3200‐0‐239973, IZSEZ0_234994; INTERREG Central Europe Programme, CE0200902.

## Conflicts of Interest

The authors declare no conflicts of interest.

## Supporting information


**Figure S1:** Time to budburst for saplings from different provenances (a) and under different transfer dates (b) in the study species.
**Figure S2:** Effect of sapling provenance, transfer date and winter warming on the time to budburst (mean ± se) for different species.
**Figure S3:** Time to budburst for saplings from different provenances under different transfer dates and winter temperature treatments across the study species.
**Figure S4:** Forcing requirements for saplings from different provenances (a) and under different transfer dates (b) for each tree species.
**Figure S5:** Forcing requirements for saplings from different transfer dates under two winter temperature treatments for each tree species.
**Figure S6:** Forcing requirements for saplings from different provenances under different transfer dates and winter temperature treatments for each tree species.
**Figure S7:** Budburst success under different provenances (a) and transfer dates (b) for each tree species.
**Figure S8:** Interactive effects of sapling provenance, transfer date, and winter temperature treatment on budburst success for each tree species.
**Figure S9:** Budburst success under different provenances, transfer dates, and winter temperature treatments for the study tree species.
**Figure S10:** Estimation of chilling and forcing requirements of the study species for each provenance.
**Figure S11:** Frost probability for each day of the year (DOY) based on long‐term historical climate data (1991–2020).
**Table S1:** Mean total number of buds per sapling and average height across species and provenances.
**Table S2:** The most parsimonious model identified for all species combined and for each tree species separately.
**Table S3:** Budburst success of saplings from different species and provenances under each transfer date.

## Data Availability

The experimental data supporting the findings of this study are openly available in the ChillingPro project dataset at Zenodo (https://doi.org/10.5281/zenodo.17780222). Climate data were derived from the E‐OBS dataset (https://www.ecad.eu/download/ensembles/download.php).
