# Supplementary material for: Provenance‐Specific Chilling and Forcing Requirements Shape Spring Phenology in Three European Temperate Tree Species
Source: Glob Chang Biol. 2026 Apr 10;32(4):e70851. doi: 10.1111/gcb.70851 (PMC13069241; doi:10.1111/gcb.70851)
Supplement: Supplementary file 1 — Figure S1: Time to budburst for saplings from different provenances (a) and under different transfer dates (b) in the study species. Figure S2: Effect of sapling provenance, transfer date and winter warming on the time to budburst (mean ± se) for different species. Figure S3: Time to budburst for saplings from different provenances under different transfer dates and winter temperature treatments across the study species. Figure S4: Forcing requirements for saplings from different provenances (a) and under different transfer dates (b) for each tree species. Figure S5: Forcing requirements for saplings from different transfer dates under two winter temperature treatments for each tree species. Figure S6: Forcing requirements for saplings from different provenances under different transfer dates and winter temperature treatments for each tree species. Figure S7: Budburst success under different provenances (a) and transfer dates (b) for each tree species. Figure S8: Interactive effects of sapling provenance, transfer date, and winter temperature treatment on budburst success for each tree species. Figure S9: Budburst success under different provenances, transfer dates, and winter temperature treatments for the study tree species. Figure S10: Estimation of chilling and forcing requirements of the study species for each provenance. Figure S11: Frost probability for each day of the year (DOY) based on long‐term historical climate data (1991–2020). Table S1: Mean total number of buds per sapling and average height across species and provenances. Table S2: The most parsimonious model identified for all species combined and for each tree species separately. Table S3: Budburst success of saplings from different species and provenances under each transfer date. [file GCB-32-e70851-s001.docx]

**Supporting Information for**

Provenance-specific chilling and forcing requirements shape spring phenology in three European temperate tree species

Zhaofei Wu^1*^, Manuel G. Walde^1^, Ilka Beil^2^, Marcin Klisz^3^, Yann Vitasse^1, 4^

**Affiliations**

^1^ Swiss Federal Institute for Forest, Snow and Landscape Research WSL, Birmensdorf, 8903, Switzerland;

^2^ Experimental Plant Ecology, University of Greifswald, Greifswald, Germany;

^3^ Dendrolab IBL, Department of Silviculture and Genetics of Forest Trees, Forest Research Institute, Poland;

^4^Oeschger Centre for Climate Change Research, University of Bern, Bern 3012, Switzerland.

*** Corresponding author:** [zhaofei.wu@wsl.ch](mailto:zhaofei.wu@wsl.ch)

**Table. S1.** Mean total number of buds per sapling and average height across species and provenances.

| **Species** | **Provenances** | **Height (cm)** | **Bud number** | |
| --- | --- | --- | --- | --- |
| *Fagus sylvatica* | Germany | 38.7 ± 11.9 | 24 ± 1.2 | 27 ± 1.0 |
|  | Poland | 71.0 ± 14.4 | 36 ±1.9 |  |
|  | Spain | 13.9 ± 5.7 | 15 ±1.1 |  |
|  | Switzerland | 58.0 ± 13.5 | 32 ±1.7 |  |
| *Quercus robur* | Germany | 67.9 ± 10.4 | 32 ±2.3 | 39 ± 1.7 |
|  | Poland | 73.9 ± 21.2 | 27 ±1.9 |  |
|  | Spain | 63.2 ± 13.9 | 55 ±4.3 |  |
|  | Switzerland | 86.9 ± 21.0 | 42 ±2.7 |  |
| *Tilia cordata* | Germany | 59.5 ± 19.2 | 25 ±1.5 | 27 ± 0.8 |
|  | Poland | 111.5 ± 19.3 | 31 ±1.6 |  |
|  | Spain | 84.5 ± 16.2 | 29 ±1.3 |  |
|  | Switzerland | 62.2 ± 14.7 | 22 ±1.1 |  |

**Table. S2. The most parsimonious model identified for all species combined and for each tree species separately.** The most parsimonious model was determined using multi-factor analysis of variance (ANOVA) and identified via stepwise model selection based on Akaike’s Information Criterion (AIC).

| **Species** | **Selected models** | **AIC** |
| --- | --- | --- |
| All species | Forcing requirement ~ Transfer date + Species + Winter warming + Provenance + Transfer date*Species + Transfer date*Winter warming + Species*Provenance + Species*Winter warming + Winter warming*Provenance | 7406 |
|  | Budburst success ~ Transfer date + Species + Winter warming + Transfer date*Species + Species*Winter warming | 2850 |
| *Quercus robur* | Forcing requirement ~ Provenance + Transfer date + Winter warming + Transfer date*Provenance + Transfer date*Winter warming | 2446 |
|  | Budburst success ~ Transfer date + Provenance | 890 |
| *Fagus sylvatica* | Forcing requirement ~ Transfer date + Provenance + Winter warming + Transfer date*Provenance + Transfer date*Winter warming | 2585 |
|  | Budburst success ~ Provenance + Transfer date + Transfer date*Provenance | 901 |
| *Tilia cordata* | Forcing requirement ~ Transfer date + Winter warming + Transfer date*Winter warming | 2207 |
|  | Budburst success ~ Transfer date + Winter warming + Transfer date*Winter warming | 961 |

**Table. S3. Budburst success of saplings from different species and provenances under each transfer date.** Budburst success was calculated as the percentage of buds that achieved budburst to the total number of buds per sapling.

| **Species** | **Transfer date** | **Germany** | | **Poland** | | **Spain** | | **Switzerland** | |
| --- | --- | --- | --- | --- | --- | --- | --- | --- | --- |
|  |  | Number | Percentage | Number | Percentage | Number | Percentage | Number | Percentage |
| ***Fagus sylvatica*** | 2024-11-05 | 6 | 100% | 6 | 100% | 6 | 100% | 6 | 100% |
|  | 2024-12-12 | 12 | 100% | 12 | 100% | 12 | 100% | 12 | 100% |
|  | 2025-01-15 | 12 | 100% | 12 | 100% | 12 | 100% | 12 | 100% |
|  | 2025-02-25 | 12 | 100% | 12 | 100% | 12 | 100% | 12 | 100% |
| ***Quercus robur*** | 2024-11-05 | 6 | 100% | 6 | 100% | 6 | 100% | 6 | 100% |
|  | 2024-12-12 | 12 | 100% | 12 | 100% | 12 | 100% | 11 | 92% |
|  | 2025-01-15 | 12 | 100% | 12 | 100% | 12 | 100% | 12 | 100% |
|  | 2025-02-25 | 12 | 100% | 12 | 100% | 12 | 100% | 12 | 100% |
| ***Tilia cordata*** | 2024-11-05 | **1** | **17%** | **1** | **17%** | **1** | **17%** | **1** | **17%** |
|  | 2024-12-12 | 11 | 92% | 8 | 67% | 11 | 92% | 9 | 75% |
|  | 2025-01-15 | 11 | 92% | 12 | 100% | 12 | 100% | 12 | 100% |
|  | 2025-02-25 | 12 | 100% | 12 | 100% | 12 | 100% | 12 | 100% |

**Note**: For *Tilia cordata* transferred into the climate chamber in November, the temperature was adjusted to 3 °C on April 10, 2025, for two weeks and subsequently to 2 °C on May 22, 2025, for three weeks to fulfill chilling requirements. Budburst then occurred between August 10 and 20, more than nine months after the onset of the forcing treatment, although all saplings remained alive (green phloem and tissue intact).


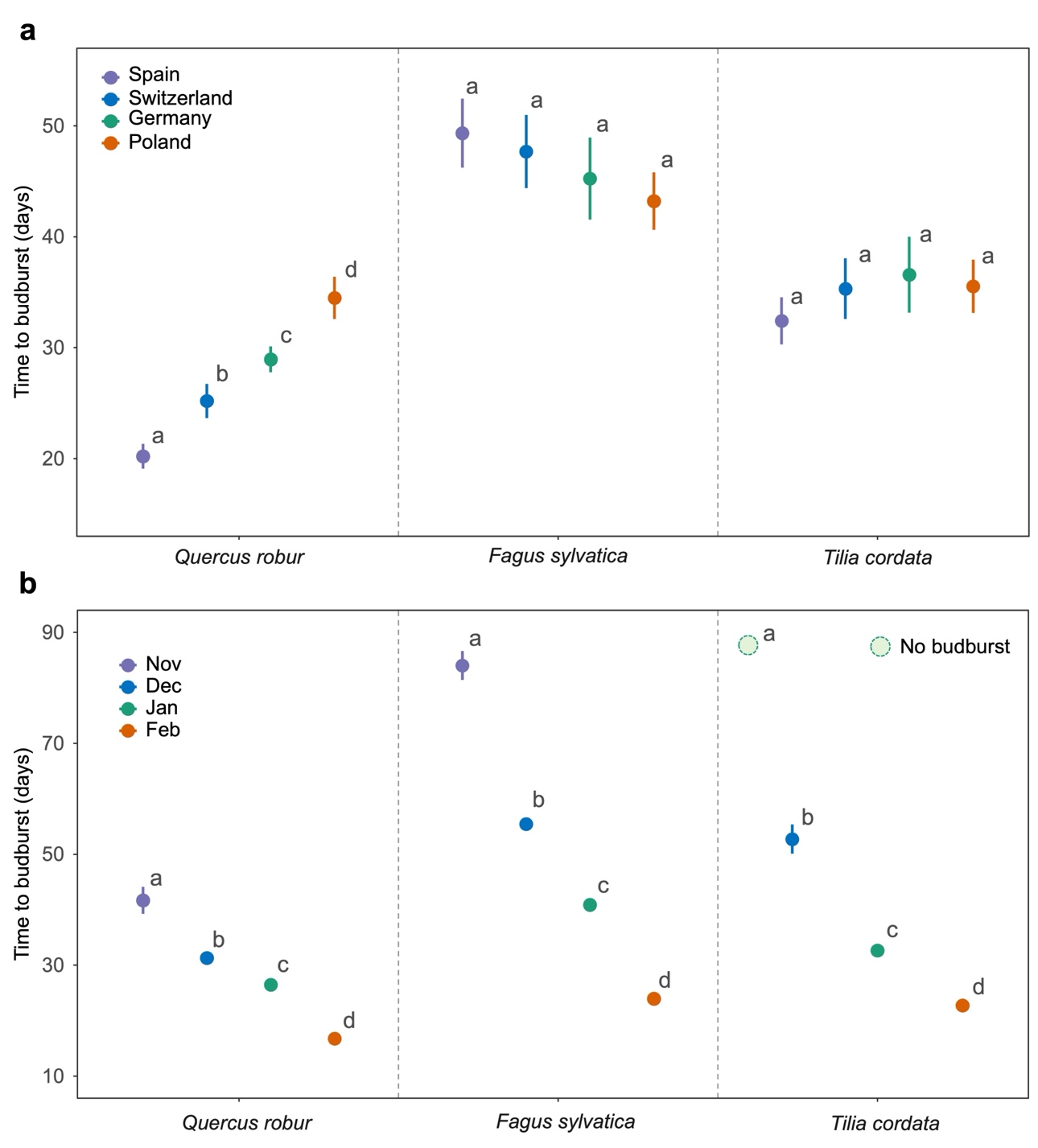


**Figure S1. Time to budburst for saplings from different provenances (a) and under different transfer dates (b) in the study species.** Time to budburst is represented as the days to budburst after being transferred to the 20°C climate chamber. Each data point represents the mean ± SE of individual saplings under each treatment. The green dashed point indicates saplings that had not yet reached budburst. Different letters denote significant differences between sapling origins based on a two-sided t-test (*P* < 0.05).


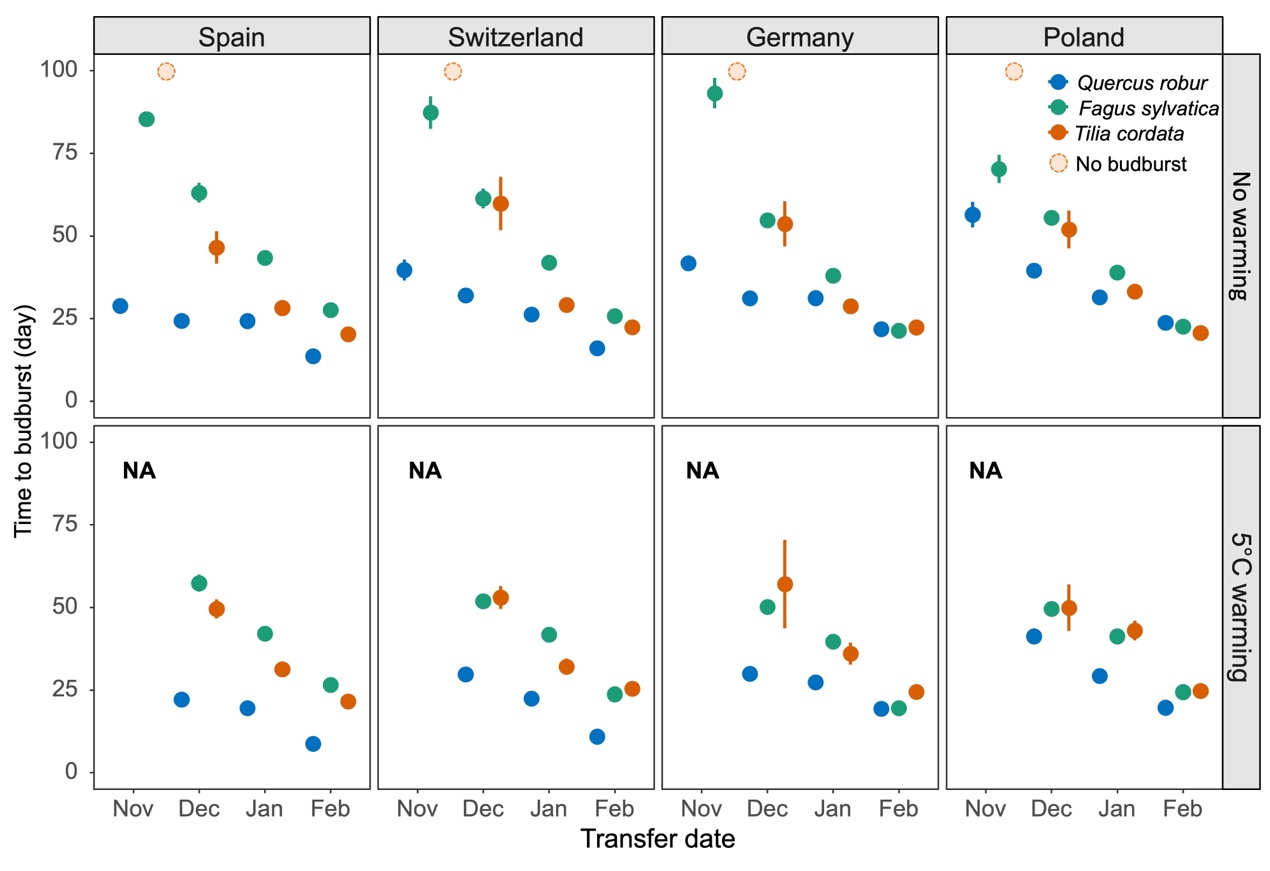


**Figure S2. Effect of sapling provenance, transfer date and winter warming on the time to budburst (mean ± se) for different species.** Time to budburst is represented as the days to budburst after being transferred to the 20°C climate chamber. The no-warming treatment indicates the open-top chambers without warming; the 5°C-warming treatment refers to the open-top chambers with a 5°C increase above ambient temperature. The orange dashed point indicates saplings that had not reached budburst.


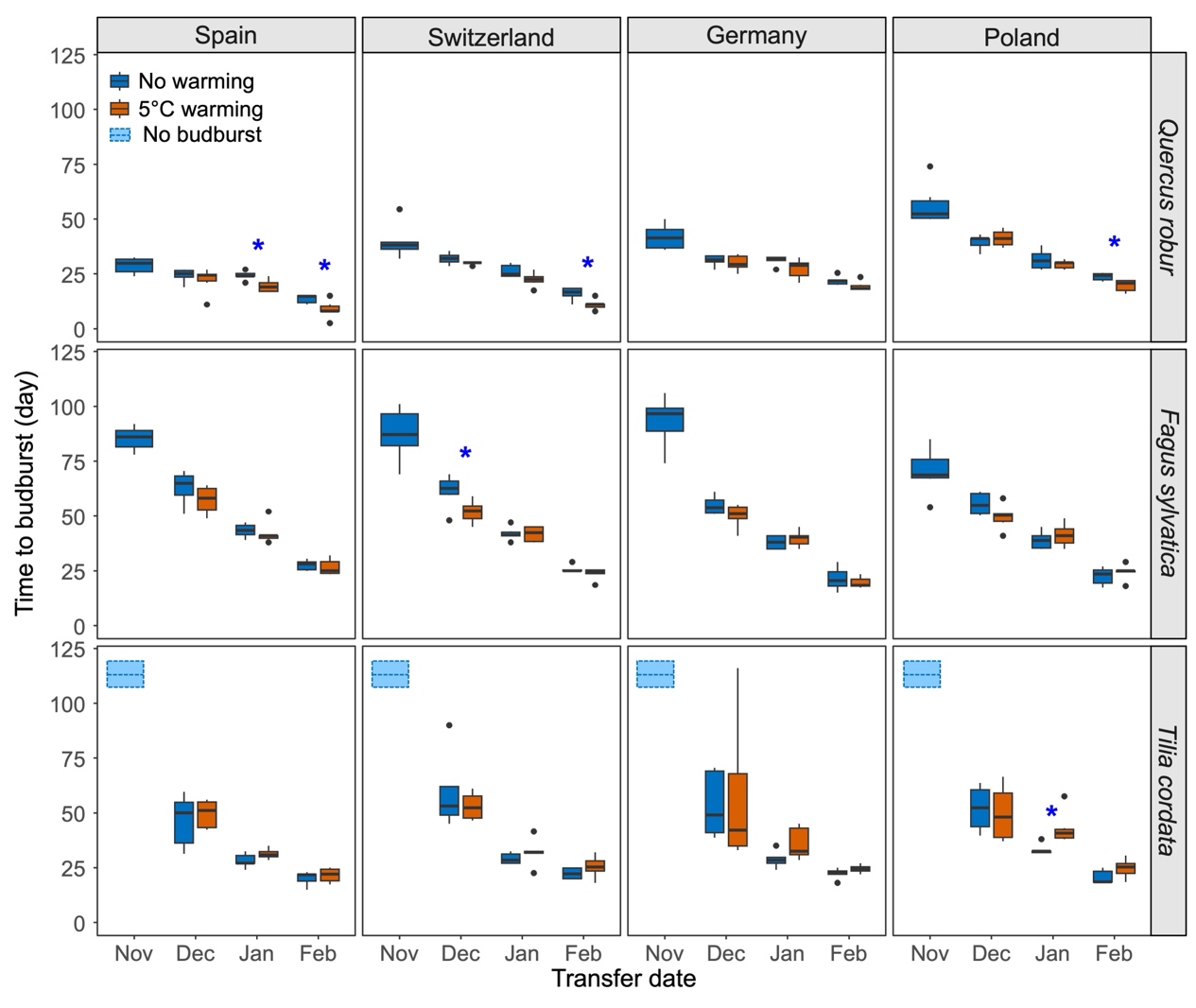


**Figure S3. Time to budburst for saplings from different provenances under different transfer dates and winter temperature treatments across the study species.** Time to budburst is represented as the days to budburst after being transferred to the 20°C climate chamber. The no-warming treatment indicates the open-top chambers without warming; the 5°C-warming treatment refers to the open-top chambers with a 5°C increase above ambient temperature. Boxplots show the first quartile, median, and third quartile, as well as the minimum and maximum values within 1.5 times the interquartile range. The blue dashed box indicates saplings that had not reached budburst. Asterisks (*) denote significant differences between winter temperature treatments based on a two-sided t-test (*P* < 0.05).

**
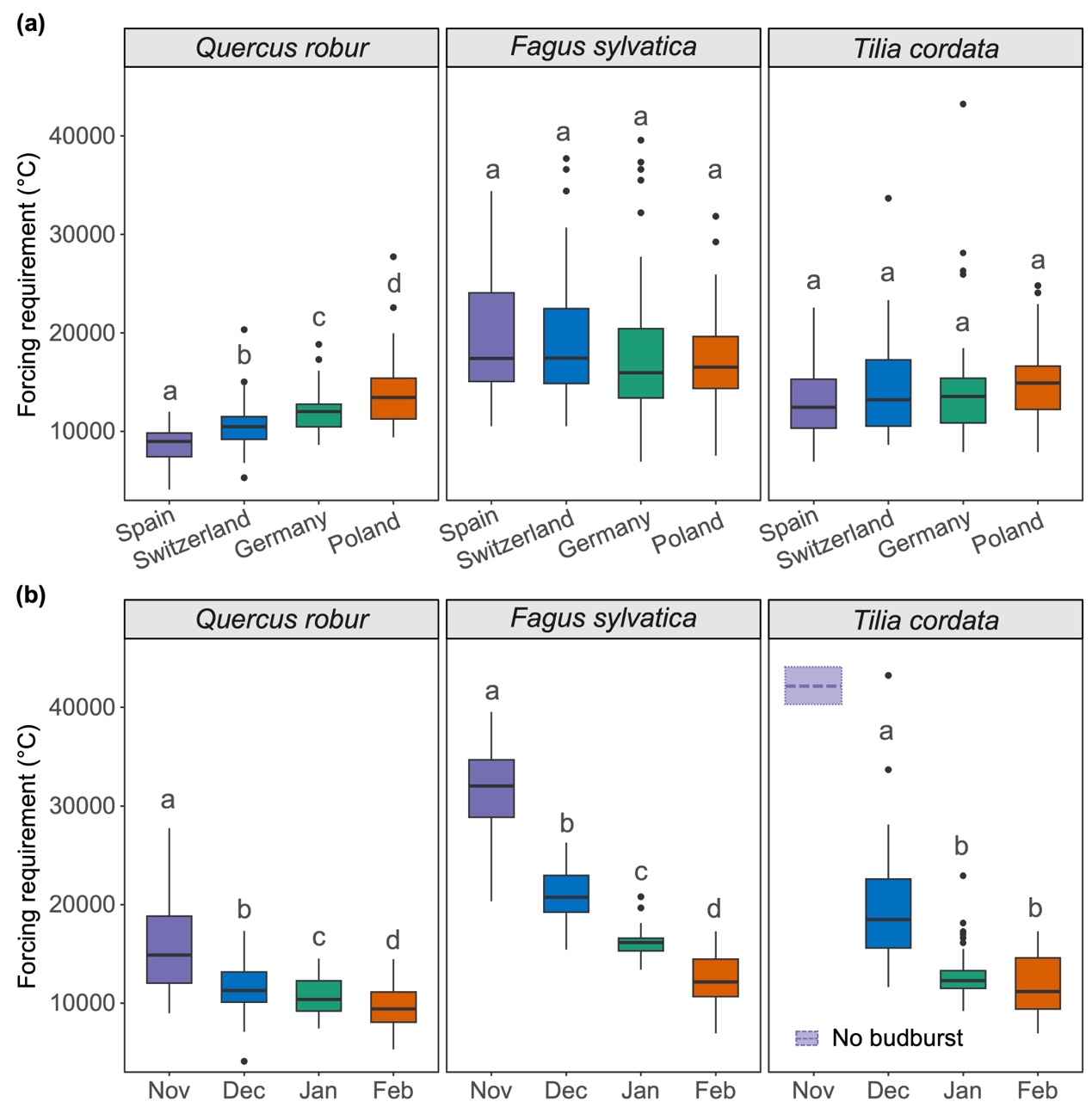
**

**Figure S4.** **Forcing requirements for saplings from different provenances (a) and under different transfer dates (b) for each tree species.** Forcing requirement is represented as growing degree hours, calculated as the cumulative temperature above 5°C. This includes the forcing accumulation in the open-top chambers from January 1, 2025, to the transfer date, as well as accumulation in the climate chambers from the transfer date until budburst. Boxplots show the first quartile, median, and third quartile, as well as the minimum and maximum values within 1.5 times the interquartile range. The purple dashed box indicates saplings that had not reached budburst. Different letters denote significant differences between winter temperature treatments based on a two-sided t-test (*P* < 0.05).


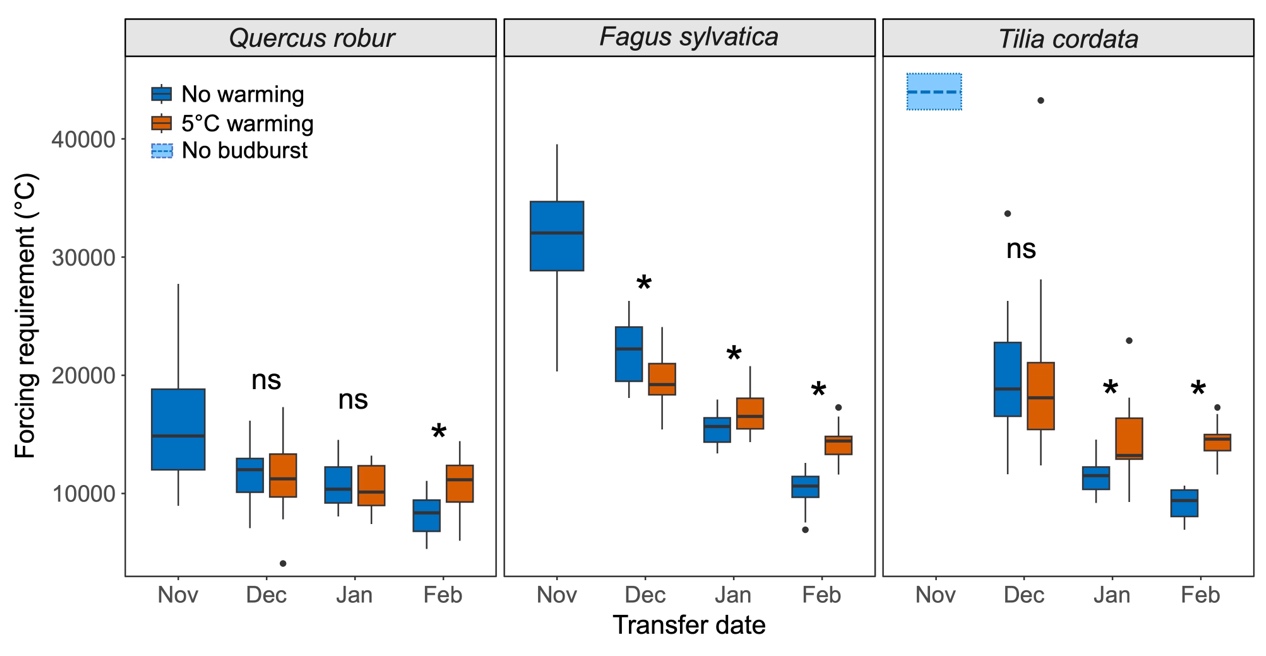


**Figure S5. Forcing requirements for saplings from different transfer dates under two winter temperature treatments for each tree species.** Forcing requirement is represented as growing degree hours, calculated as the cumulative temperature above 5°C. This includes the forcing accumulation in the open-top chambers from January 1, 2025, to the transfer date, as well as accumulation in the climate chambers from the transfer date until budburst. The no-warming treatment indicates the open-top chambers without warming; the 5°C-warming treatment refers to the open-top chambers with a 5°C increase above ambient temperature. Boxplots show the first quartile, median, and third quartile, as well as the minimum and maximum values within 1.5 times the interquartile range. The blue dashed box indicates saplings that had not reached budburst. Asterisks (*) denote significant differences between winter temperature treatments based on a two-sided t-test (*P* < 0.05); ns indicates no statistically significant difference.


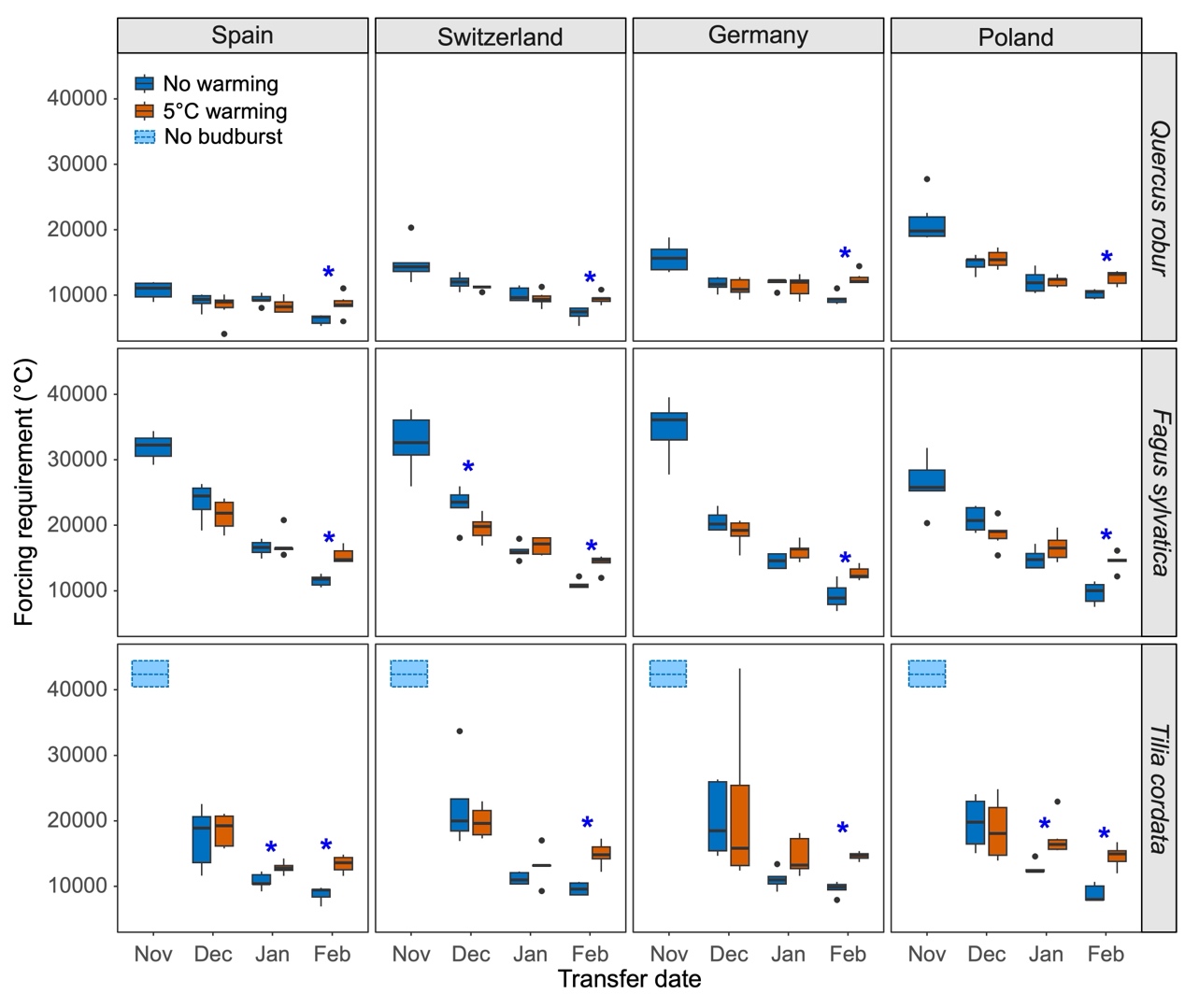


**Figure S6. Forcing requirements for saplings from different provenances under different transfer dates and winter temperature treatments for each tree species.** Forcing requirement is represented as growing degree hours, calculated as the cumulative temperature above 5°C. This includes the forcing accumulation in the open-top chambers from January 1, 2025, to the transfer date, as well as accumulation in the climate chambers from the transfer date until budburst. The no-warming treatment indicates the open-top chambers without warming; the 5°C-warming treatment refers to the open-top chambers with a 5°C increase above ambient temperature. Boxplots show the first quartile, median, and third quartile, as well as the minimum and maximum values within 1.5 times the interquartile range. The blue dashed box indicates saplings that had not reached budburst. Asterisks (*) denote significant differences between winter temperature treatments based on a two-sided t-test (*P* < 0.05).

**
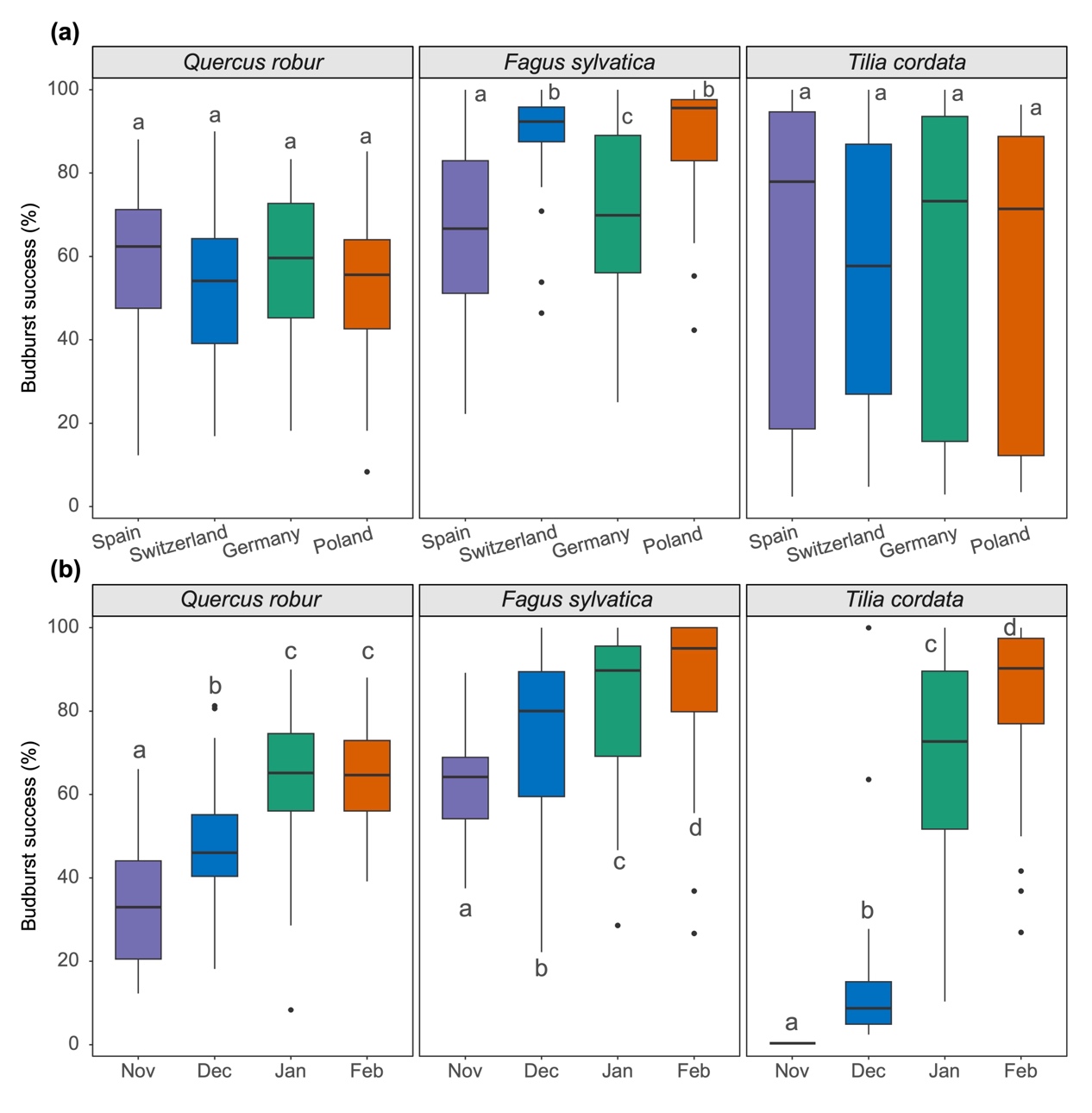
**

**Figure S7. Budburst success under different provenances (a) and transfer dates (b) for each tree species.** Budburst success was calculated as the percentage of buds that achieved budburst to the total number of buds per sapling. Boxplots show the first quartile, median, and third quartile, as well as the minimum and maximum values within 1.5 times the interquartile range. Different letters denote significant differences between winter temperature treatments based on a two-sided t-test (*P* < 0.05).


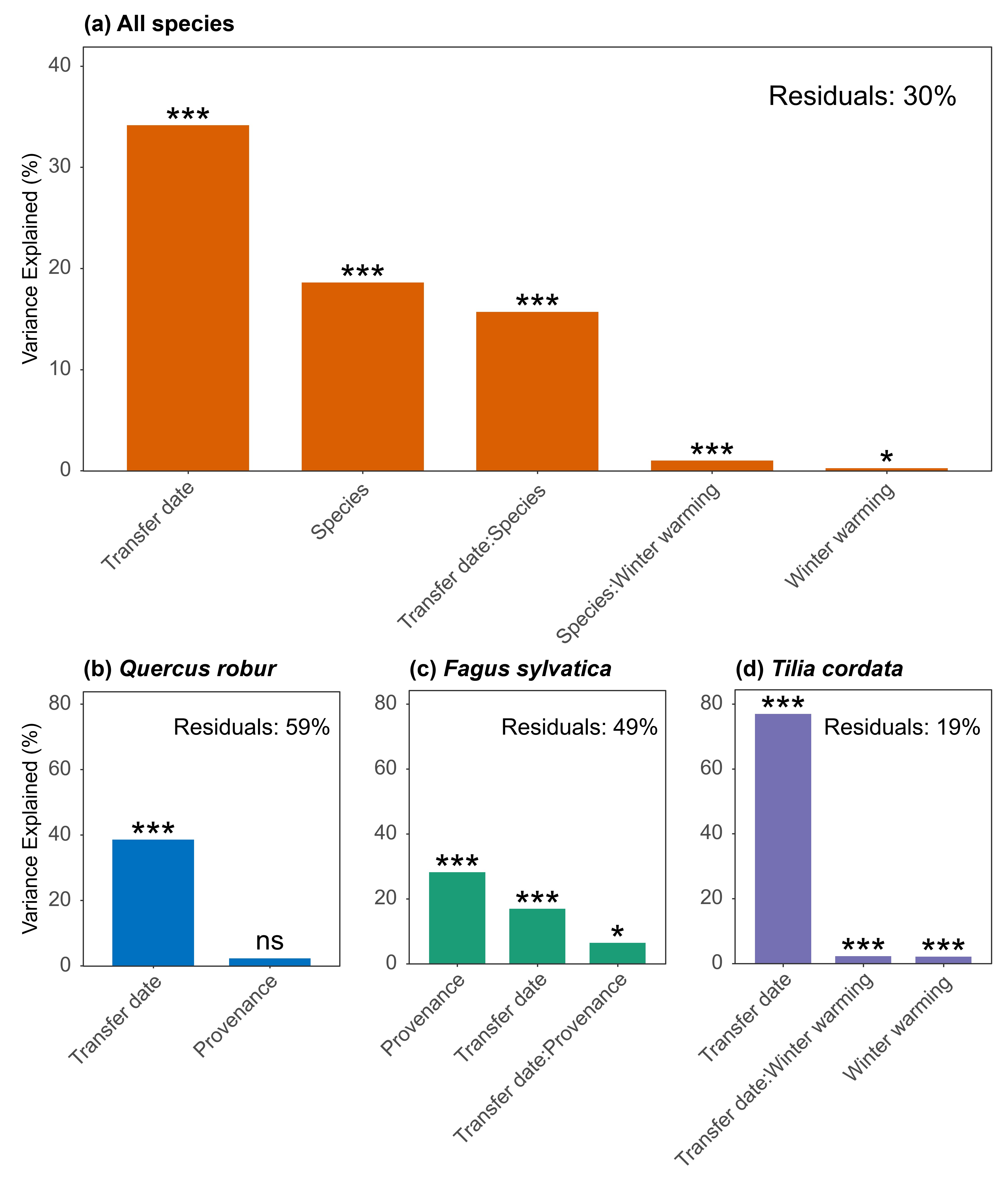


**Figure S8. Interactive effects of sapling provenance, transfer date, and winter temperature treatment on budburst success for each tree species.** Budburst success was calculated as the number of buds that had burst divided by the total number of buds. The contribution of each variable to budburst success was assessed using a multi-factor analysis of variance (ANOVA), with budburst success as the response variable and the interaction among sapling provenance, transfer date, winter temperature treatment, and tree species as explanatory variables. The most parsimonious model was identified via stepwise model selection based on Akaike’s Information Criterion (AIC), starting from a null model. The residual represents the proportion of variance not explained by the selected variables. Asterisks indicate statistical significance based on F-tests from the ANOVA: *** *P* < 0.001; ** *P* < 0.01; * *P* < 0.05.


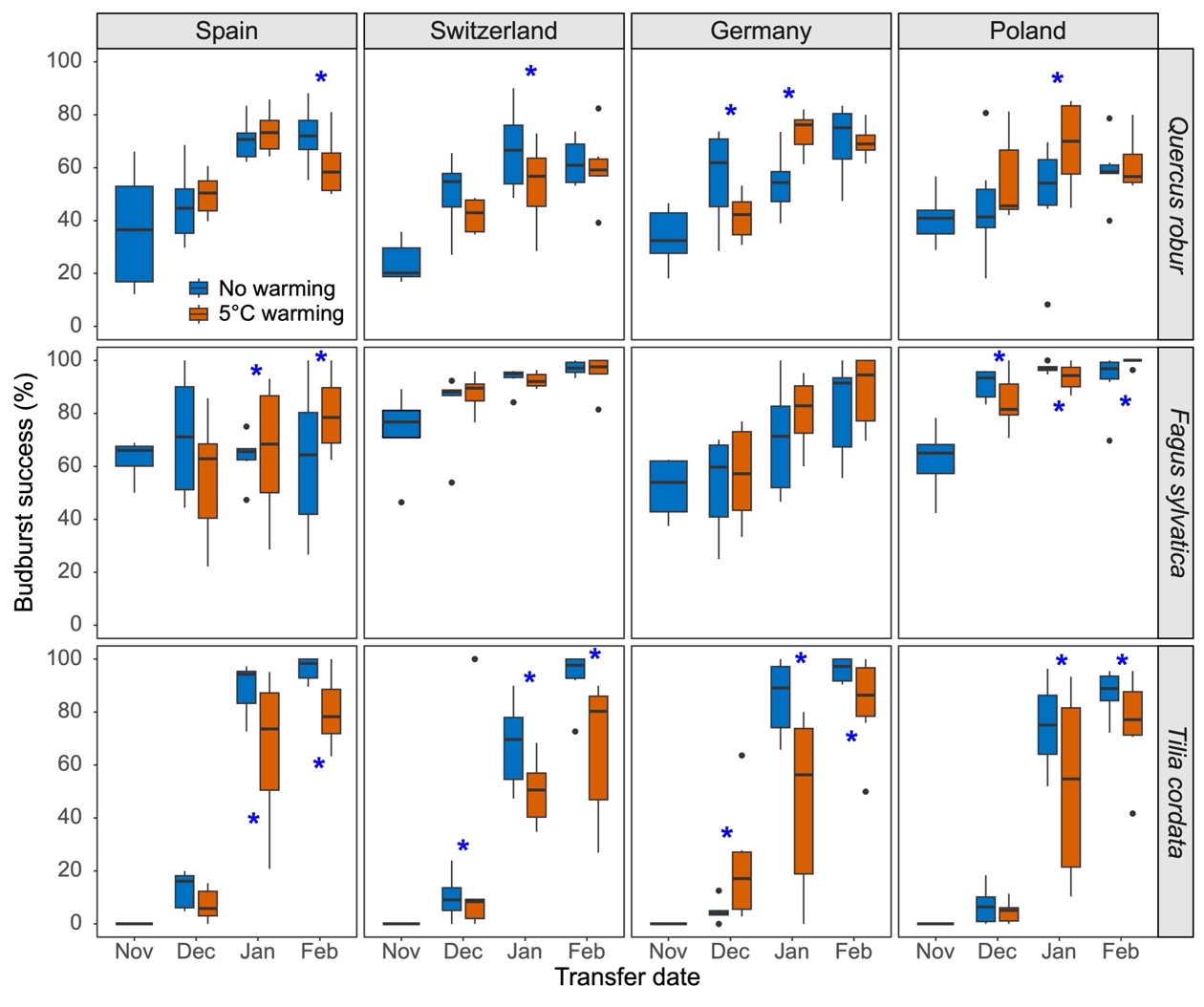


**Figure S9. Budburst success under different provenances, transfer dates, and winter temperature treatments for the study tree species.** Budburst success was calculated as the number of buds that had burst divided by the total number of buds. The no-warming treatment indicates the open-top chambers without warming; the 5°C-warming treatment refers to the open-top chambers with a 5°C increase above ambient temperature. Boxplots show the first quartile, median, and third quartile, as well as the minimum and maximum values within 1.5 times the interquartile range. Asterisks (*) denote significant differences between winter temperature treatments based on a two-sided t-test (*P* < 0.05), other pairwise comparisons were not statistically significant.


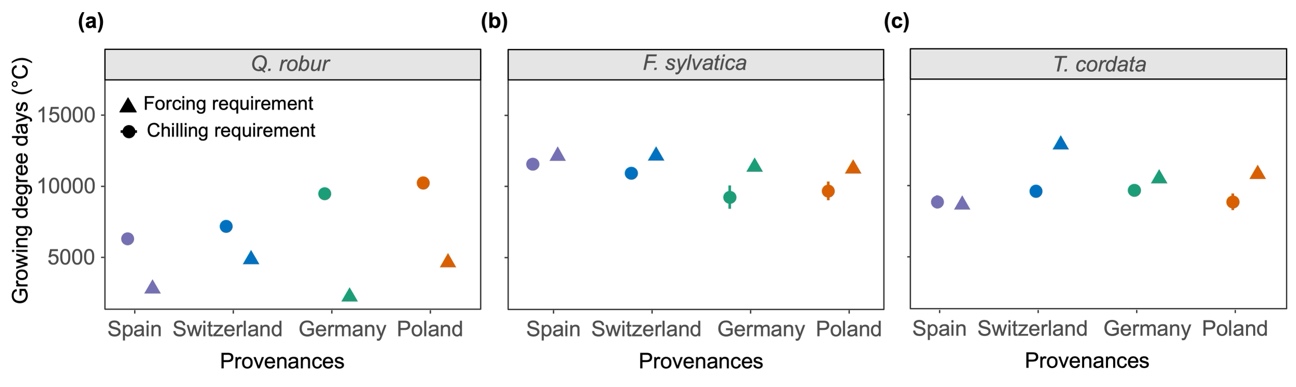


**Figure S10. Estimation of chilling and forcing requirements of the study species for each provenance.** Forcing requirement is expressed by the amount of growing degree hours required to budburst for the saplings transferred in February (maximum chilling exposure), whereas chilling requirement is expressed by the difference in forcing between the February and December transfers.


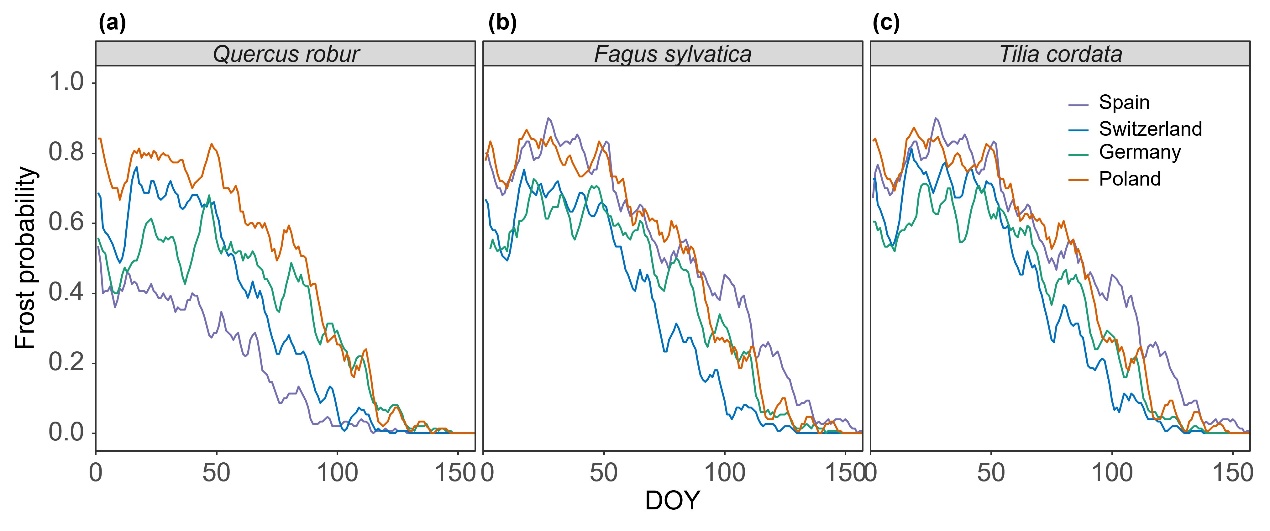


**Figure S11. Frost probability for each day of the year (DOY) based on long-term historical climate data (1991–2020).** Frost was defined as days when the minimum temperature fell below 0 °C. Frost probability was smoothed using a 5-day moving window.
